# The spatial distribution characteristics and influencing factors of key villages in rural tourism in China

**DOI:** 10.1371/journal.pone.0330486

**Published:** 2025-08-19

**Authors:** Jiqiang Niu, Hao Guo, Weichun He, Feng Xu, Guangyu Zhang

**Affiliations:** 1 School of Geographical Sciences, Xinyang Normal University, Xinyang City, Henan Province, China; 2 Henan Key Technology Engineering Research Center of Microwave Remote Sensing and Resource Environment Monitoring, Xinyang City, Henan Province, China; Peking University, CHINA

## Abstract

Rural tourism key villages in China serve as critical nodes driving the development of rural tourism. Based on a dataset of 1,195 key villages, this study employs Ripley’s *K*-function and geographic detector methods to investigate their spatial distribution patterns and influencing factors. The findings reveal: ① The spatial distribution of rural tourism key villages demonstrates a strong directional alignment toward major cities and tourist attractions; ② Significant regional disparities exist in aggregation scales, with the eastern region exhibiting a substantially higher concentration than the central and western regions; ③ Both natural environmental factors and socio-economic factors influence rural tourism key villages, with road network density, river network density, and per capita disposable income exerting the most pronounced effects. These results provide valuable theoretical insights and practical guidance for the differentiated development and rural revitalization of key villages in China’s rural tourism sector.

## 1. Introduction

Against the backdrop of rapid urbanization, rural decline has become a global challenge, particularly acute in China [1]. In response, the 19th National Congress of the Communist Party of China introduced the rural revitalization strategy, aimed at unlocking rural development potential, fostering high-quality rural growth, and promoting urban-rural integration. The rural revitalization industry serves as a cornerstone, with ecological preservation as its safeguard. As an emerging sector, rural tourism has gained prominence as a “golden industry” due to its extensive industrial linkages, economic benefits, and minimal environmental impact [2]. rural tourism key villages act as “testing grounds” for resource integration and as “pioneers” in advancing rural revitalization. Establishing distinctive tourism villages and driving high-quality rural development have become vital pathways to achieving rural revitalization. Therefore, understanding the spatial distribution, patterns, and influencing factors of China’s rural tourism key villages is critical for advancing rural tourism and revitalization.

The rural territorial system is a complex and dynamic entity [3], which has undergone substantial structural transformation throughout its long historical evolution [4]. This evolution has produced a distinctive landscape in which large-scale village decline coexists with the emergence and strengthening of key villages. In response to this phenomenon, scholars have carried out extensive research on various types of villages to uncover the broader patterns and mechanisms underlying rural development [5,6]. For instance, Chinese scholar Li Xiaojian and others have emphasized that future rural development in China will inevitably involve the decline of certain villages, while others will evolve into new functional forms such as large-scale grain production bases, residential-oriented settlements, specialized agricultural hubs, and tourism-oriented villages. Among these, villages with distinctive characteristics-such as traditional villages, ethnic minority villages, forest villages, and key villages for rural tourism-have been identified as “growth poles” in the broader context of rural transformation due to their unique resource endowments, cultural heritage, and industrial development models. These villages have attracted growing scholarly attention, leading to a substantial body of research and theoretical advancement in this field [7–9].

As a vital component of modern tourism, rural tourism has emerged as a popular means for individuals to relax, reconnect with nature, and seek nostalgic experiences. It is a developmental outcome of economies reaching a certain level of maturity, with its origins tracing back to Europe. Over time, rural tourism has expanded significantly in both developed and developing countries [10,11], evolving into an essential driver of rural revitalization and urban–rural integration. International studies have demonstrated that rural tourism facilitates the inflow of capital, talent, information, and technology into rural areas, thereby revitalizing rural economies, enhancing the quality of rural living environments, upgrading infrastructure, restructuring spatial configurations, and ultimately promoting sustainable rural development [12]. In the context of China’s rapid urbanization, rural tourism has experienced substantial growth [13]. As key carriers of rural tourism development, the spatial distribution characteristics and influencing factors of key villages for rural tourism have attracted increasing scholarly attention.

At present, most scholars use the data of a single batch of rural tourism focus to study the distribution of a single batch of rural tourism focus villages, while we have organized the data of three consecutive batches and explored the spatial and temporal changes in dynamics of the three batches. In terms of research scale, existing studies have been carried out mainly on a national scale or in special provinces (e.g., Zhejiang, Jilin, etc.), and the spatial pattern characteristics of key villages of rural tourism in different economic development contexts [14,15]. On the basis of existing studies, this paper further introduces the perspective of multi-scale analysis, and systematically compares the spatial agglomeration characteristics of China’s key villages for rural tourism at the national scale, provincial scale, and regional scales in the east, middle and west. The horizontal comparison of the degree of agglomeration at different scales reveals the differences in the macro spatial pattern of key villages for rural tourism, reflecting the heterogeneity of spatial structure formed by the influence of uneven regional development. This multi-scale analysis framework helps to deepen the understanding of the spatial agglomeration mechanism of rural tourism, and provides a more scientific and operable theoretical support and policy reference for the formulation of rural tourism development strategies that are tailored to the needs of local conditions and categories. Regarding methodological approaches, researchers have employed techniques such as standard deviation ellipse analysis, kernel density estimation, spatial autocorrelation, correlation analysis, and geographical detectors to investigate the spatial distribution and underlying drivers of key rural tourism villages [16,17]. In this study, Ripley’s function analysis is introduced as a complementary means to investigate the degree of agglomeration and spatial distribution patterns of key rural tourism villages at different scales. This method can reveal the clustering or dispersion characteristics of point data at different spatial scales, which can help to identify the spatial heterogeneity of key rural tourism villages and their differentiated distribution patterns at local and regional scales. Compared with the traditional global spatial autocorrelation method, the introduction of Ripley’s function not only expands the technical path of rural tourism spatial pattern research, but also enhances the ability to analyze the formation mechanism of agglomeration phenomenon to a certain extent, and improves the depth and precision of spatial analysis. Content-wise, the literature has largely addressed topics including regional disparities, spatial hotspots, development potential, and functional zoning, as well as the relationships between the distribution of key rural tourism villages and natural or socio-economic conditions [18,19]. Meanwhile, when analyzing the influencing factors by using geodetectors, we systematically sorted and compared the core indicator data of the three batches of key villages of rural tourism in different years, combined with the multi-temporal and multi-batches of panel data, to enhance the robustness and scientificity of the analysis. By introducing the time dimension, we not only enhance the credibility of causal inference, but also effectively reduce the interference of chance factors and extreme values on the results, further improve the representativeness and universality of the results, and provide a more solid empirical support for revealing the dominant driving mechanism behind the development of rural tourism. From a comprehensive point of view, although existing studies have explored the spatial pattern of key villages in China’s tourism, the excavation of the spatial distribution characteristics of key villages in rural tourism at different spatial scales has yet to be strengthened, especially in the exploration of the driving effect and spatial radiation mechanism between key cities, core scenic spots and key villages of different types of rural tourism, the current research is still insufficient. Existing research focuses on the description of the spatial pattern at the macro level, and generally lacks a systematic analysis of the multilevel and multiscale linkage between cities, scenic spots and villages, and has not yet revealed the actual mechanism of its role in the integration of regional tourism resources, the reconstruction of transportation networks and the coordinated development of urban and rural areas. In view of this, future research should focus on the quantitative identification and modeling analysis of the spatial gravitational effect of the core nodes, the radiation path and its dynamic evolution process, in order to further deepen the understanding and mastery of the rural tourism spatial organization system and the mechanism of regional coordinated development.

Building on this foundation, this study examines the three batches (2019, 2020, and 2021) of China’s rural tourism key villages designated by the Ministry of Culture and Tourism of the People’s Republic of China. Employing methods such as kernel density estimation, Ripley’s function analysis, and geographic detectors, the research focuses on the clustering and spatial distribution characteristics of rural tourism key villages across the eastern, central, and western regions. Furthermore, it delves into the influencing factors shaping their spatial distribution, providing a theoretical basis and practical guidance for the development of rural tourism key villages and advancing rural revitalization in China.

## 2. Materials and methods

In this study, kernel density analysis, geographic concentration index, Ripley’s *L*-function analysis and geodetector method are used to reveal the spatial and temporal distribution characteristics and driving mechanisms of key villages of rural tourism in China from multiple perspectives. These methods are often used by scholars to study the spatial distribution problem. Firstly, to explore the spatial distribution characteristics of key villages of rural tourism, we should understand the agglomeration of the research object in different regions, so the advantage of the geographic concentration index is that it can numerically quantitatively measure the degree of agglomeration of the key villages of rural tourism in different regional perspectives; secondly, we want to study the spatial distribution characteristics of the key villages of rural tourism in the overall scope of the research area. Secondly, we want to refine the distribution characteristics of key rural tourism villages in the overall study area in terms of spatial and temporal patterns, and the kernel density estimation method can more intuitively show the dense or sparse areas of the study object in geospatial space, which makes up for the shortcomings of the geographic concentration index that can not be visualized; and then we use Ripley’s *L*-function to detect the aggregation patterns in different spatial scales, which makes up for the limitations of the kernel density estimation that can only reflect the overall trend. The Ripley’s *L* function is then used to detect aggregation patterns at different spatial scales, which compensates for the fact that the kernel density estimation can only reflect the overall trend; finally, we want to study which factors have an impact on the formation of key villages in rural tourism, and since the factors affecting the distribution pattern of key villages in rural tourism are multifaceted and multifactorial, we choose to analyze the driving factors and their interactions behind spatial heterogeneity in-depth by means of a geodetector. The four methods complement each other to achieve the comprehensive research goal from distribution visualization, pattern quantification to mechanism analysis.

### 2.1. Geographical concentration index

Geographic concentration index is used to reflect whether the indicator is concentrated or dispersed in a certain area, the larger the value of *G*, the higher the concentration of the indicator; the smaller the value of *G*, the lower the concentration of the indicator [20]. The formula is:


$$G=100\times \sqrt{\sum_{i=1}^{n}{\left(\frac{P_{i}}{Q}\right)}^{2}}$$ (1)

where *G* denotes the geographic concentration index, *P* denotes the number of samples in a certain region, *Q* denotes the sum of samples, and *n* denotes the number of different regions. In the calculation of geographic concentration index, this paper takes the spatial area of each region as a reference benchmark for uniform distribution, and considers that in an ideal state the research object should be evenly distributed according to the proportion of regional area, i.e., the expected share of each region is a_i_/A. The actual distribution of the index will be compared with this mean value, and then the degree of its spatial concentration will be measured. In this research, if the 1,195 key villages of rural tourism are uniformly distributed in the three major zones of the east, center and west, the value of *G* is 59.917; if they are uniformly distributed in the provinces (cities) and autonomous regions, the value of *G* is 18.300; and if they are uniformly distributed at the level of the municipal area (including provincial municipalities), the value of *G* is 7.703. At different scales, the ratio of the geographic concentration index of the key villages of rural tourism to that of the uniformly distributed state is larger, and the ratio of the geographic concentration index of the key villages of rural tourism to that of the uniformly distributed state is larger. The larger the ratio of the concentration index, the more obvious the degree of geographic concentration in the distribution of rural tourism key villages [21].

### 2.2. Kernel density estimation

Kernel density estimation is a nonparametric method. Kernel density analysis is easy to implement and can better reflect the ground distance decay effect in the spatial distribution of geographical phenomena [22]. Its calculation formula is:


$$f(x)=\frac{1}{nh}\sum_{i=1}^{n}k\left(\frac{x-x_{i}}{h}\right)$$ (2)

where: $k\left(\frac{x-x_{i}}{h}\right)$ is the kernel function; *h* is the distance decay threshold (i.e., bandwidth); and $x-x_{i}$ is the distance from the rural tourism focus village *x* to the measurement marker village $x_{i}$ [23]. Among them, the bandwidth settings are initially set based on the Silverman empirical method [24], and combined with the geographic scale and spatial distribution characteristics of the study area, the parameter values that are most suitable for portraying the agglomeration pattern are selected through a multi-group bandwidth sensitivity analysis, so as to ensure that the estimation results are balanced between smoothing and spatial information retention. In this paper, the kernel density analysis module in ArcGIS 10.8 is used to perform density analysis of POI data to identify the main and secondary centers of rural tourism key villages in China. In addition, the kernel density estimation method was used to analyze the spatial distribution of the elements in the rural tourism key villages in China in the agglomeration area, and to analyze the spatial agglomeration characteristics of the elements in different cities [25]. In this research, a Gaussian kernel function (Gaussian kernel) is used in the kernel density estimation, which possesses good smoothing and stability, and is suitable for expressing the characteristics of the continuous distribution of the research object in geospace. In addition, to ensure that the choice of kernel function does not cause excessive bias to the results, the Epanechnikov kernel is also referenced for comparative analysis, and the results remain consistent, verifying the robustness of the estimation.

### 2.3. Ripley’s *L* function analysis

Ripley’s *K* function is a commonly used method for point pattern analysis, with the greatest advantage of analyzing spatial patterns at multiple scales [26]. By using Ripley’s *K* function, the point data set is analyzed for the degree of clustering at different distances. The calculation formula is as follows:


$$K\left(d\right)=A\sum_{i}^{n}\sum_{j}^{n}\frac{W_{ij}\left(d\right)}{n^{2}}$$ (3)

$$L\left(d\right)=\sqrt{\frac{K\left(d\right)}{\pi }}-d$$ (4)

where: *A* is the area of the study area; *n* is the number of sample points; *d* is the observation scale; *W*_*ij*_(*d*) is the distance between two sample points *i* and *j* within the observation scale *d* [27]. Besag proposed to utilize *L*(*d*) instead of *K*(*d*) in order to ensure the stability of the variance, so this study adopts the deformation of the Ripley’s *K* function [28], General Through the Monte Carlo method to simulate the function of the confidence interval, if the function value is greater than the upper limit of the confidence interval, the sample points show significant agglomeration characteristics, if the function value is less than the lower limit of the confidence interval, the sample points show significant uniform distribution characteristics, if the function value is located within the confidence interval indicates that the sample points show a random distribution characteristics. In order to comprehensively understand the scale of agglomeration in different regions, we have examined the agglomeration characteristics of the key villages of Chinese rural tourism in the eastern, central and western regions at multiple scales, so as to illustrate the range of spatial scales for their location choices [29].

### 2.4. Geodetector

Geodetectors have been used primarily to analyze the degree of explanation of various influences and multifactor interactions. This is mainly because the geodetector *q*-value has a clear physical meaning, there is no linearity assumption, and objectively detects that the independent variable explains 100 × *q*% of the dependent variable, especially because the dependent variable *Y* and the independent variable *X* in this paper are numerical quantities, and after the discretization of *X* is converted into a type of quantities, the relationship between *Y* and *X* established by using the geodetector will be more reliable than other regressions. Therefore, we used factor detection and interaction detection in the geodetector [30] model to identify the main influencing factors and their interaction relationships that affect the spatial pattern of key villages in rural tourism in China [31]. The dependent variable *Y* in the model of this study is the number of key villages of rural tourism in different cities, and the independent variable *X* is the natural environmental factors and socio-economic factors affecting key villages of rural tourism. Factor detection mainly analyzes the degree of explanation of different influencing factors on the spatial pattern of key villages of rural tourism in China, and a larger *q* value indicates that the independent variable *X* has a stronger explanatory power on the dependent variable *Y*, and vice versa. The formula is:


$$\begin{array}{c} q=1-\frac{SSW}{SST},\\ SSW=\sum_{h=1}^{L}N_{h}\sigma_{h}^{2},SST=N\sigma^{2} \end{array}$$ (5)

where *SSW* and *SST* are the sum of the intra-stratum variance and the total variance of the whole area, respectively, *N*_*h*_ and *N* are the number of cells in stratum *h* and the whole area, respectively; $\sigma_{h}^{2}$ and $\sigma^{2}$ are the variance of the *Y*-values in stratum *h* and the whole area, respectively.

Interaction detection focuses on identifying whether the explanatory power of the dependent variable is enhanced or weakened when different independent variables act together. Firstly, the *q*-values of the 2 dependent variables $X_{1}$ and $X_{2}$ on *Y* are calculated separately, $q(X_{1})$ and $q(X_{2})$ respectively, then the *q*-values when these 2 factors interact, i.e., $q(X_{1}\cap X_{2})$, and finally the interaction type is determined by comparing $q(X_{1})$, $q(X_{2})$ and $q(X_{1}\cap X_{2})$ [32] (Table 1).

**Table 1:** Judgment criteria and interaction type between two covariates.

Type	Judgment criteria
Nonlinear attenuation	$q(X_{1}\cap X_{2})<\min\left(q\left(X_{1}\right),q\left(X_{2}\right)\right)$
One-factor nonlinear attenuation	$\min\left(q\left(X_{1}\right),q\left(X_{2}\right)\right)<q(X_{1}\cap X_{2})<\max\left(q\left(X_{1}\right),q\left(X_{2}\right)\right)$
Two-factor enhancement	$q\left(X_{1}\cap X_{2}\right)>\max\left(q\left(X_{1}\right),q\left(X_{2}\right)\right)$
Independent	$q(X_{1}\cap X_{2})=q\left(X_{1}\right)+q\left(X_{1}\right)$
Nonlinear enhancement	$q(X_{1}\cap X_{2})>q\left(X_{1}\right)+q\left(X_{1}\right)$

The formation of rural tourism key villages is influenced by both natural geographic factors and socio-economic conditions, with the two interacting to promote the development of rural tourism villages in a given region [33]. In this study, the number of rural tourism key villages in a city is treated as the dependent variable (*Y*), while natural factors are represented by the average annual precipitation (*X*_1_), average annual temperature (*X*_2_), and river network density (*X*_3_). Socio-economic factors are represented by per capita GDP (*X*_4_), per capita disposable income (*X*_5_), the proportion of the tertiary sector in GDP (*X*_6_), and road network density (*X*_7_) (Table 2). These variables are selected to explore the determinants behind the spatial patterns of rural tourism key villages. Previous research indicates that both natural and socio-economic factors play a significant role in the formation of these villages over the long term [34–37]. Consequently, this study uses the three-year average of the data prior to the announcement of rural tourism key villages for regression analysis. Due to the unavailability of data for certain autonomous regions and areas, these were excluded from the study. Descriptive statistical analysis of the data reveals that the mean value significantly exceeds the standard deviation, indicating a lower degree of dispersion and making the data more suitable for regression analysis [38,39].

**Table 2:** Selection of influencing factors.

Descriptive statistics	2019		2020		2021	
	Average value	Standard deviation	Average value	Standard deviation	Average value	Standard deviation
Annual precipitation(°C)	4.9936	15.0336	5.2456	14.8969	14.9998	5.8352
Average annual temperature(mm)	175.3987	1115.9250	144.2747	1208.1025	1086.5044	432.0815
River network density(km/km^2^)	0.8525	0.7382	0.8525	0.7382	1.0993	0.7380
GDP per capita(yuan)	68807	69605.5842	70334	69034.2521	69688	35527.0339
Per capita disposable income(yuan)	33075	36144.7012	34301	37465.3268	26822	15739.7833
Tertiary sector as a share of GDP(%)	0.4600	0.6144	0.4470	0.6132	0.4831	0.0869
Road density(km/km^2^)	0.8525	0.7382	0.8525	0.7382	1.1011	0.7361

## 3. Data sources

**Fig 1:**
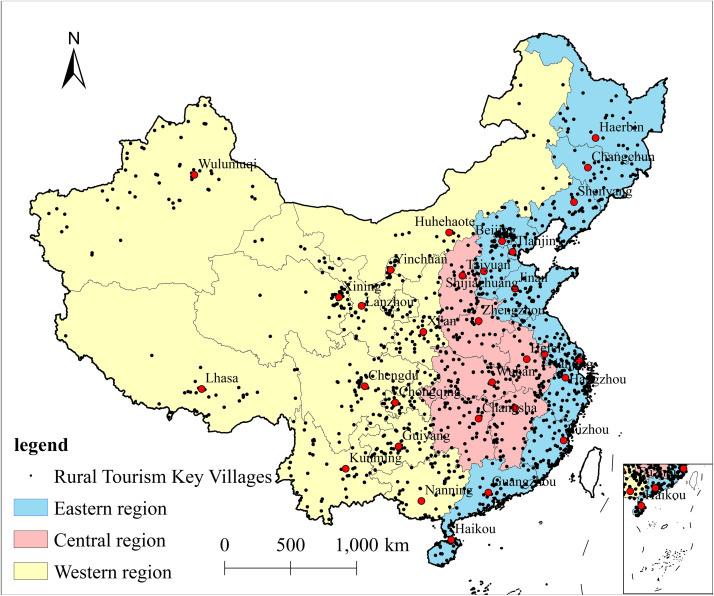
Spatial pattern of rural tourism key villages in China. Revision No.: GS(2019)1822.

Taking the three batches (2019, 2020 and 2021) of China’s rural tourism key villages (https://sjfw.mct.gov.cn/site/dataservice/rural) recognized by the Ministry of Culture and Tourism of the People’s Republic of China as the research object (excluding Hong Kong, Macao and Taiwan Provinces), the crawler was applied to crawl the full names of all 1199 The full names of all 1199 Chinese rural tourism key villages and 318 5A-level tourism scenic spots were crawled using crawlers, and Baidu map was used to pick up the coordinate system to obtain the latitude and longitude coordinates of the tourism key villages and 5A-level tourism scenic spots, among which 4 tourism key villages were named after the construction corps, and it was not possible to obtain the coordinates, so a total of 1,195 samples of tourism key villages were involved in the present study (Fig 1), which formed the basic data for the spatial analysis. The statistical data were obtained from the China Urban Statistical Yearbook (2020–2022) published by the National Bureau of Statistics of China, and some missing data were filled in through the statistical yearbooks of each province (city).

## 4. Results and analysis

### 4.1. Distribution characteristics of key villages for rural tourism at different spatial scales

The geographic concentration index (GCI) of rural tourism key villages was calculated at different spatial scales. The results showed that at the three major regional scales, the geographic concentration index of key villages for rural tourism was 59.917, which was 1.039 times higher than that under a uniform distribution state (G = 57.677); At the provincial level, the geographic concentration index of key villages for rural tourism is 18.300, which is 1.019 times higher than that in a uniformly distributed state (G = 17.961); At the urban scale, the geographic concentration index of key rural tourism villages is 7.703, which is 1.482 times higher than that of the uniformly distributed state (5.198). Overall, the rural tourism key villages show a spatial pattern of dispersion at large scales and aggregation at small scales. For example, the distribution is relatively balanced at the three major zone scales and the provincial scale, and the distribution pattern is more concentrated at the municipal scale as the scale narrows.

**Fig 2:**
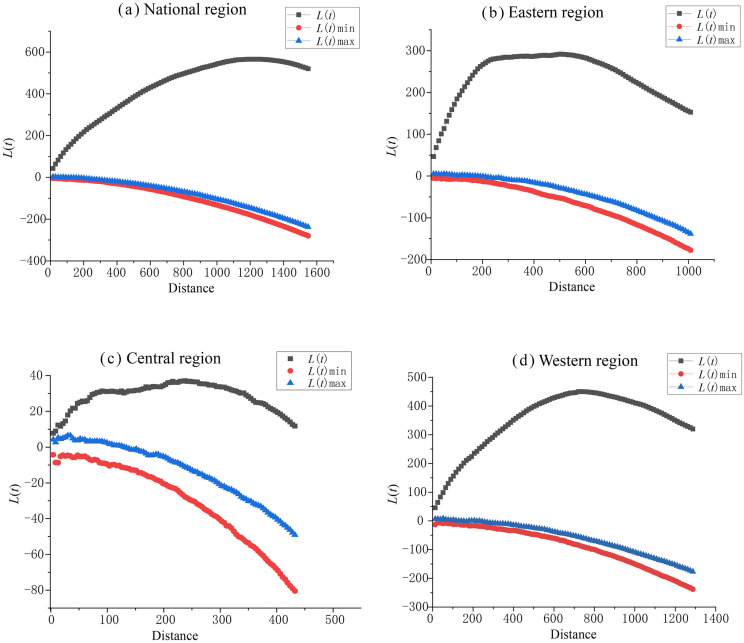
Plot of Ripley’s *L*-function values as a function of observation scale for key villages of rural tourism in different regions.

The clustering characteristics of rural tourism key villages in the three major regions were further examined using Ripley’s *L*-function analysis in Crimestat software (Fig 2). The simulation results indicate that the *L*-function curves for all three regions lie above L_max_, suggesting a clustered distribution pattern for the rural tourism key villages. Specifically, the eastern region shows the highest clustering intensity of 291.72 at a distance of 504.43 km, while the central region exhibits the maximum clustering intensity of 37.13 at 237.80 km, and the western region reaches a maximum clustering intensity of 450.13 at 733.60 km. Notably, the clustering scales and intensities in the eastern and western regions are significantly higher than those in the central region. This disparity can be attributed to the higher levels of economic development and population density in the eastern region, which stimulate greater demand for rural tourism, thus providing favorable conditions for its development. In contrast, the western region, characterized by its mountainous plateaus and ethnic minority populations, has developed unique natural and cultural landscapes, fostering the growth of distinctive rural tourism key villages. In comparison, the central region, with its moderate economic development and status as a major agricultural production base in China, features vast plains and low hills. Consequently, the distribution of rural tourism key villages is more evenly spread across the central region, with notable concentrations in specific areas such as the Dabie Mountain range, western Hebei, and western Hunan.

### 4.2. Characteristics of spatial association between key villages of rural tourism and big cities

**Fig 3:**
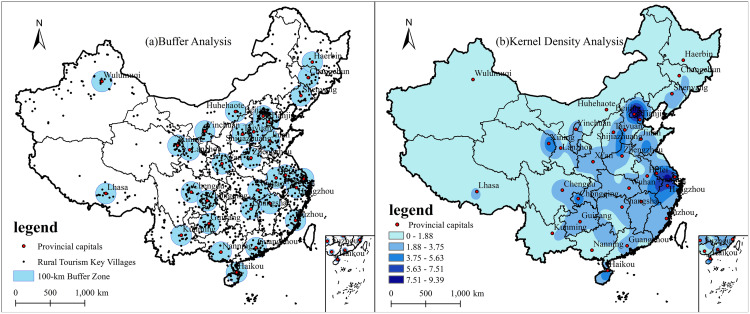
Spatial distribution of key rural tourism villages in relation to large cities based on buffer zone and kernel density analysis. Revision No.: GS(2019)1822.

Cities serve as important sources of rural tourism [40,41]. Using Chinese provincial capitals and larger cities as centers, and drawing on previous studies regarding the travel radius for leisure tourism from large urban areas [42], a 100 km buffer zone was established (Fig 3a). The analysis reveals that 390 rural tourism key villages are located within this buffer zone, accounting for approximately 32.6% of the total number of rural tourism key villages in China. This proportion is significantly higher than the ratio of the buffer zone area to the overall rural land area, indicating a clear spatial tendency of rural tourism key villages toward major urban centers. Further analysis was conducted using ArcGIS 10.8 to examine the kernel density of rural tourism key villages in China (Fig 3b). The results show that the kernel density values for these villages are notably higher around large cities, particularly in Beijing, Tianjin, Shanghai, and Hangzhou. Over time, the distribution density of rural tourism key villages in proximity to major cities has increased at a rate significantly higher than the national average. For instance, in 2019, the distribution density of the first batch of rural tourism key villages in Beijing and Shanghai was 5.5 and 7.4 per 10,000 km^2^, respectively, far exceeding the national average of 0.3 per 10,000 km^2^ during the same period. By 2019 and 2020, the density for the first two batches of rural tourism key villages increased to 19.5 and 21.1 per 10,000 km^2^. respectively, further outpacing the national average of 1.0 per 10,000 km^2^. By the time the distribution density of all rural tourism key villages was calculated, it reached 23.16 and 27.30 per 10,000 km^2^. respectively, which was considerably higher than the national average of 1.2 per 10,000 km^2^. From a comprehensive perspective, the spatial distribution of key villages for rural tourism demonstrates a strong tendency toward agglomeration around major cities. This is particularly evident in the high concentration of key villages in areas adjacent to large cities, such as provincial capitals, whereas regions located farther away from these urban centers exhibit a relatively sparse distribution.

In the context of China’s rapid urbanization, rural areas-unlike the structured and idealized urban environment-offer greater appeal to urban residents, serving as important destinations for stress relief, nature immersion, and recreation [43]. The higher levels of economic development and population density in major cities generate substantial tourism demand, forming a stable tourist base that drives the growth of rural tourism in surrounding areas. This dynamic serves as a critical foundation for the emergence of key villages for rural tourism in these regions. Furthermore, the outskirts of large cities benefit from well-developed and diverse transportation infrastructure, providing convenient access to nearby rural destinations and further facilitating tourism development. In addition, proximity to major cities enables rural areas to secure greater financial investment and attract skilled human resources, thereby enhancing the development potential and service quality of rural tourism assets [13,44]. The dual forces of a vast urban tourist market and robust infrastructure have catalyzed the rapid transformation of rural industries and landscapes in peri-urban areas [45,46], leading to a significantly higher number and density of key villages for rural tourism in these zones compared to more remote regions.

### 4.3. Characteristics of spatial association between key villages of rural tourism and important tourist attractions

**Fig 4:**
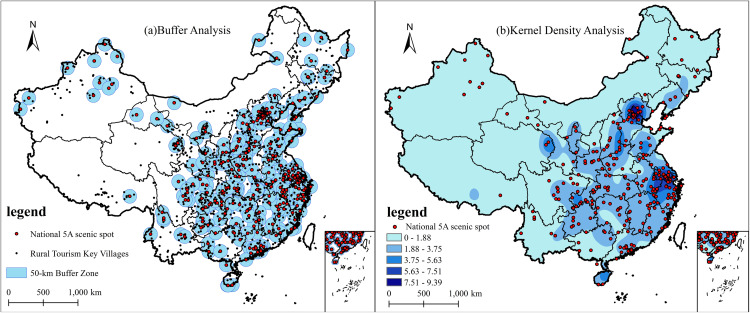
Spatial distribution relationship between rural tourism key villages and 5A-level tourist attractions based on buffer zone and kernel density analysis. Revision No.: GS(2019)1822.

Key tourist attractions are important tourist destinations for tourists traveling and have a radiation-driven role in the formation of key villages for rural tourism. Using China’s 5A-level tourist attractions as the focal point, a buffer zone with a 50 km radius was established (Fig 4a). The analysis revealed that 601 rural tourism key villages are located within this buffer zone, accounting for 50.29% of the total number of rural tourism key villages in China. Kernel density analysis (Fig 4b) indicates that the kernel density values of these villages are significantly higher in areas with a dense concentration of national 5A-level tourist attractions, while the density is lower in regions where such attractions are sparse. These findings suggest that the spatial distribution of rural tourism key villages in China exhibits a clear alignment with major scenic spots.

Amid the rapid development of the tourism economy, individual key villages for rural tourism often struggle to generate substantial appeal for tourists-particularly international visitors. However, the spatial clustering of such villages near nationally designated 5A-level scenic attractions enables them to benefit from the positive externalities associated with these high-profile destinations. The mature and stable tourist market attracted by key scenic spots provides a consistent flow of visitors to nearby rural villages. More importantly, rural tourism villages located in close proximity to 5A-level attractions are more likely to be integrated into well-established tourism routes, which not only reduces development costs through infrastructure sharing but also facilitates the formation of composite tourism products that combine “scenic sightseeing” with “rural experiences,” thereby promoting sustainable rural tourism development [13,47].

For example, located in Xining City, Qinghai Province, Datong Hui Tu Autonomous County, Shuo Bei Tibetan Township, Dong Zhi Gou village, the village is far away from the city, located in the mountainous and hilly areas, the transportation is relatively closed, the village’s tourist attraction to tourists is also very limited, but due to the proximity of the Thar Temple Scenic Area, the village has been integrated into the Thar Temple Scenic Area around the tourist routes. There are also China’s Ministry of Culture and Tourism and other departments announced the national rural tourism boutique line in the Yangtze River Delta Jiangnan water town line connecting Jiangsu Zhouzhuang, Zhejiang Wuzhen, Shanghai Zhujiajiao and other ancient towns with the surrounding rural tourism key villages, the formation of the “ancient towns + idyllic” experience belt; Xiangxi Tujia Miao hometown style line to Zhangjiajie Wulingyuan as the core, the radiation of the Phoenix Ancient City, eighteen holes, such as the village and other ethnic characteristics of the villages; Zhejiang, Anhui, Fujian, Jiangxi ecological tourism line covering Huangshan Mountain, Wuyi, Wuyi, Fujian and Gan ecological tourism lines, covering Huangshan Mountain, Wuyi, Wuyi, Wuyi and other areas. Tourism line covering Mount Huangshan, Wuyi Mountain, Mount Sanqingshan and other key scenic spots, linkage of Anhui Hongcun, Zhejiang Kaifa and other rural tourist spots. The formation of these tourism lines provides a good foundation for the development of rural tourism.

### 4.4. Influencing factors of spatial distribution of rural tourism key villages in China

The independent and dependent variables were classified using the best natural breakpoint method, transforming them into categorical variables. Subsequently, geo-detector analysis was employed to assess the spatial heterogeneity of China’s rural tourism key villages from 2019 to 2021 [48]. The p-values for all variables in the period of 2019–2021 passed the significance test, and the q-values were positive, indicating a positive correlation between these factors and the spatial distribution of rural tourism key villages (Table 3).

**Table 3:** Results of the geographical detector q value for key villages from 2019 to 2021.

First-level indicator	Secondary indicator	2019	2020	2021
Natural environmental factors	Annual precipitation(*X*_1_)	0.10	0.11	0.16
	average annual temperature(*X*_2_)	0.19	0.20	0.27
	River network density(*X*_3_)	0.34	0.47	0.54
Socioeconomic factors	GDP per capita(*X*_4_)	0.14	0.18	0.20
	Per capita disposable income(*X*_5_)	0.05	0.11	0.16
	Tertiary sector as a share of GDP(*X*_6_)	0.07	0.10	0.18
	road density(*X*_7_)	0.35	0.52	0.59

Among the natural factors, river network density (*X*_3_) has the greatest impact on the distribution of rural tourism key villages, with values of 0.34 in 2019, 0.47 in 2020, and 0.54 in 2021. This is followed by the local average annual temperature (*X*_2_), with values of 0.19 in 2019, 0.20 in 2020, and 0.27 in 2021. Additionally, average annual precipitation also demonstrates a significant positive correlation across all three years.

Most of China lies between the subtropical and temperate zones, where a higher average temperature contributes to greater climate comfort, while increased precipitation and river network density enhance water resources. These factors are favorable for agricultural development and human settlement. Consequently, areas with a comfortable climate and dense river networks often have early origins of civilization, which have fostered unique natural and cultural landscapes, providing a solid natural foundation for the formation of rural tourism key villages. Among the socio-economic factors, road network density (*X*_7_) has the most significant influence on the distribution of rural tourism key villages, with values of 0.35 in 2019, 0.52 in 2020, and 0.59 in 2021. This is followed by GDP per capita (*X*_4_), with values of 0.14 in 2019, 0.18 in 2020, and 0.20 in 2021. Per capita disposable income for urban and rural residents (*X*_5_) and the share of the tertiary sector in GDP (*X*_6_) also show significant positive correlations across all three years.

The development of rural tourism is intrinsically linked to the local economic environment and infrastructure. Higher levels of economic development contribute to a larger potential tourist market, while greater road network density improves accessibility to rural areas, both of which are crucial for the growth of rural tourism. Therefore, areas with a high level of economic development and dense road networks tend to have a larger number of rural tourism key villages.

**Fig 5:**
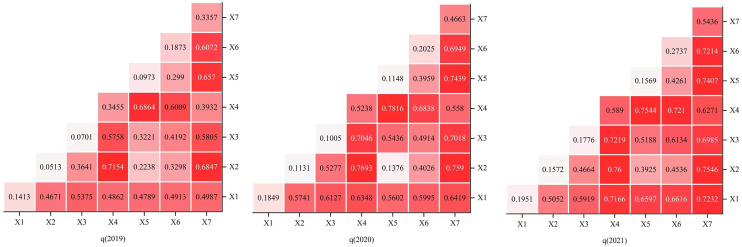
Heat map of the interaction of various influential factors on the spatial distribution of key rural tourism villages in China, 2019–2021.

To further explore the interaction effects between different factors, interaction detection in GeoDetector was employed to analyze the degree of interaction response across different years (Fig 5). The results of the interaction detection reveal that the driving force of the two-factor interactions is stronger than that of any individual factor, with the interaction among factors exhibiting a nonlinear enhancement. The interaction effects between two factors contribute significantly to the spatial distribution of rural tourism key villages, with no factors appearing independent or weakening the overall impact.

Specifically, the interaction between per capita disposable income (*X*_5_), road network density (*X*_7_), river network density (*X*_3_), and average annual precipitation (*X*_1_) plays a crucial role in shaping the spatial distribution of rural tourism key villages. Among these interactions, the combination of per capita disposable income (*X*_5_) and road network density (*X*_7_) demonstrates the strongest driving force, with three-year q-values of 0.7154, 0.7693, and 0.7600, respectively. The second most influential interaction is between per capita disposable income (*X*_5_) and river network density (*X*_3_), with q-values of 0.6847, 0.7590, and 0.7546, respectively, followed closely by the interaction between average annual precipitation (*X*_1_) and road network density (*X*_7_), with q-values of 0.6864, 0.7693, and 0.7600 over the three years. Per capita disposable income reflects the spending capacity of tourists in rural tourism key villages, contributing to a high-quality customer base. Road network density facilitates travel, enhancing the feasibility of choosing tourism routes. River network density and annual precipitation ensure adequate water resources, providing a solid foundation for the development of rural tourism key villages. These factors collectively create favorable conditions for the growth of rural tourism [49,50]. Additionally, the interaction between average annual precipitation (*X*_1_) and per capita disposable income (*X*_5_) exhibits a weaker driving force on the spatial distribution of rural tourism villages, with q-values of 0.2238, 0.1376, and 0.3925 over the three years. The weaker impact of this interaction is likely due to the relatively independent nature of these two variables, which lack a strong correlation.

## 5. Conclusion

This study employs Ripley’s *L*-function analysis to examine the degree of agglomeration of rural tourism villages at different spatial scales. Subsequently, buffer analysis and kernel density estimation are applied to investigate the relationship between rural tourism villages and large cities, as well as national 5A-level tourist attractions. Finally, seven indicators from natural environmental and socio-economic factors are selected for in-depth mechanistic analysis using GeoDetector. The key findings are as follows:

(1) The rural tourism key villages in China exhibit a relatively weak degree of spatial agglomeration at the level of the three major zones and at the provincial scale. However, as the spatial scale narrows to the municipal level, the degree of agglomeration becomes more pronounced, reflecting a spatial pattern characterized by relative dispersion at the provincial scale and relative concentration at the municipal scale. In terms of agglomeration magnitude and intensity, both are significantly higher in the eastern and western regions compared to the central region.
(2) The number and density of rural tourism key villages in China surrounding provincial capital cities (including municipalities directly under the central government) and national 5A-level tourist attractions are significantly higher than in other regions. The spatial distribution of these villages shows a clear orientation towards major cities and prominent tourist destinations.
(3) The spatial distribution of rural tourism key villages in China is influenced by both socio-economic and natural environmental factors. Among these, the density of the local road network, river network density, and per capita disposable income have the most significant impact on the distribution of these villages. Furthermore, the intensity of these factors’ effects shows a consistent increasing trend. Interaction detection results indicate that the majority of these interactions exhibit nonlinear enhancement, with some showing a two-factor enhancement. Notably, the interaction between per capita disposable income and road and river network density is the most pronounced, while the interaction between annual precipitation and per capita disposable income is relatively weaker. However, the interaction factor analysis also reveals an overall trend of enhancement.

Against the backdrop of China’s comprehensive rural revitalization and the integrated development of culture and tourism, this study investigates the spatial distribution and influencing factors of key villages for rural tourism across the country. By employing a suite of spatial analysis methods, the study offers a multi-scale examination-encompassing zonal, provincial, and municipal levels-of the degree and intensity of agglomeration among key villages. It further elucidates the spatial relationships between these villages and major cities as well as national scenic attractions, while quantitatively assessing the driving factors that shape their spatial patterns. In contrast to previous studies, this research emphasizes the micro-scale distribution characteristics and typological evolution of key villages, thereby extending the theoretical framework of rural tourism spatial structures and their formation mechanisms. It also contributes to the integration of rural geography and tourism geography as intersecting academic fields. The findings of this study not only deepen theoretical understanding of the spatial dynamics of rural tourism development in China but also provide a valuable reference for urban tourism planning and rural tourism resource integration in the context of contemporary rural transformation.

## 6. Discussion

As China’s economy continues to grow and living standards improve, rural tourism has emerged as a significant option for leisure and vacation. Key villages for rural tourism, by leveraging their natural resources and locational advantages, have shown strong development potential and become important instruments for implementing the rural revitalization strategy at multiple governmental levels. In this context, the present study explores the spatial distribution characteristics and influencing factors of key rural tourism villages in China. However, limitations in data completeness and accessibility hinder the construction of a comprehensive and logically consistent indicator system, particularly in capturing multidimensional drivers such as social capital, cultural attractiveness, and transportation accessibility, thereby constraining the systematic analysis of spatial differentiation mechanisms. Moreover, macro-level analyses based solely on statistical data are insufficient to reveal the complex local dynamics shaping the formation of rural tourism villages and fail to reflect spatial heterogeneity across different types and regions. To address these issues, future research will focus on four key areas: integrating big data and traditional statistical data to improve indicator systems and better capture the effects of social and cultural factors; conducting comparative analyses across different geographic environments (e.g., plains vs. mountainous regions, developed vs. underdeveloped areas) to provide tailored development strategies; strengthening field-based case studies to uncover the internal mechanisms behind the evolution of different village types (e.g., suburban leisure-oriented, industry-driven); and advancing interdisciplinary approaches by incorporating perspectives from geography, economics, sociology, and ecology to provide a more holistic understanding of rural tourism development.

## Supporting information

S1 File Renamed_2bb82. (PDF)

S2 File Renamed_6e706. (PDF)

S3 File Renamed_79a0c. (PDF)

S4 File Renamed_dd228. (PDF)
