# Supplementary material for: The spatial distribution characteristics and influencing factors of key villages in rural tourism in China
Source: PLoS One. 2025 Aug 19;20(8):e0330486. doi: 10.1371/journal.pone.0330486 (PMC12364373; doi:10.1371/journal.pone.0330486)
Supplement: S1 File — (PDF) [file pone.0330486.s001.pdf]

ID				经度	纬度
1	北京市	怀柔区渤海镇北沟村	村庄	116.5446	40.42989
2		延庆区井庄镇柳沟村	村庄	116.0925	40.44436
3		密云区古北口镇古北口村	村庄	117.1575	40.69228
4		房山区周口店镇黄山店村	村庄	115.8559	39.68901
5		怀柔区喇叭沟门满族乡中榆树店村	村庄	116.5832	40.92474
6		门头沟区斋堂镇灵水村	旅游景点	115.7278	40.00132
7		顺义区龙湾屯镇柳庄户村	村庄	116.8691	40.19451
8		延庆区刘斌堡乡姚官岭村	村庄	116.1937	40.54647
9		门头沟区斋堂镇马栏村	村庄	115.6886	39.9359
10	天津市	蓟州区下营镇常州村	村庄	117.5104	40.21576
11		蓟州区渔阳镇西井峪村	村庄	117.4109	40.07106
12		蓟州区下营镇郭家沟村	村庄	117.4796	40.18793
13		蓟州区穿芳峪镇小穿芳峪村	村庄	117.5363	40.10246
14		蓟州区穿芳峪镇毛家峪村	村庄	117.521	40.09568
15		蓟州区穿芳峪镇大巨各庄村	村庄	117.5674	40.07801
16		蓟州区上仓镇程家庄村	村庄	117.3742	39.91024
17	河北省	石家庄市平山县岗南镇李家庄村	村庄	114.0054	38.37371
18		邯郸市馆陶县寿山寺乡寿山寺东村	村庄	115.2149	36.54737
19		衡水市武强县周窝镇周窝村	村庄	115.886	37.99598
20		保定市涞水县三坡镇百里峡村	旅游景点	115.3835	39.65842
21		张家口市蔚县暖泉镇西古堡村	村庄	114.4272	39.80033
22		雄安新区雄县张岗乡王村	村庄	116.2065	39.00521
23		唐山市曹妃甸区十里海养殖场	村庄	118.4809	39.15884
24		邢台市沙河市柴关乡王硇村	村庄	114.0571	36.908
25		保定市竞秀区江城乡大激店村	休闲娱乐	115.3827	38.83308
26		石家庄市正定县正定镇塔元庄村	村庄	114.5214	38.15452
27		秦皇岛市北戴河区北戴河村	村庄	119.418	39.86019
28	山西省	晋中市昔阳县大寨镇大寨村	村庄	113.7131	37.56904
29		吕梁市汾阳市贾家庄镇贾家庄村	村庄	111.8181	37.29294
30		阳泉市平定县娘子关镇娘子关村	乡镇	113.8683	37.96019
31		长治市上党区振兴新区振兴村	村庄	113.1243	35.89429
32		忻州市岢岚县宋家沟乡宋家沟村	村庄	111.6735	38.64405
33		晋城市城区北石店镇司徒村	村庄	112.8883	35.53107
34		晋中市平遥县段村镇横坡村	村庄	112.1744	37.07114
35		临汾市乡宁县关王庙乡坂儿上村	餐饮	111.0445	35.77896
36	蒙古自治	巴彦淖尔市临河区狼山镇富强村	村庄	107.3947	40.84065
37		呼伦贝尔市额尔古纳市蒙兀室韦苏木室韦	乡镇	119.73	50.81324
38		鄂尔多斯市乌审旗无定河镇巴图湾村	村庄	108.7882	37.97916
39		赤峰市喀喇沁旗西桥镇雷家营子村	村庄	118.8841	41.85837
40		呼和浩特市新城区保合少镇恼包村	村庄	111.8984	40.89407
41		兴安盟乌兰浩特市义勒力特镇义勒力特嘎	村庄	122.0225	46.17047
42		包头市土默特右旗沟门镇西湾村	村庄	110.5117	40.59616
43		呼伦贝尔市鄂伦春自治旗大杨树镇多布库尔猎	村庄	124.5303	49.76169
44		通辽市科左后旗散都苏木车家窝堡村	村庄	123.3283	43.0611
45	辽宁省	丹东市凤城市凤山区大梨树村	村庄	123.9576	40.40005
46		沈阳市沈北新区石佛寺街道石佛一村	村庄	123.3432	42.13122

47		大连市旅顺口区水师营街道小南村	村庄	121.2544	38.84662
48		本溪市本溪满族自治县小市镇同江峪村	村庄	124.0497	41.29737
49		锦州市凌海市翠岩镇牤牛屯村	村庄	120.998	41.22535
50		阜新市细河区四合镇黄家沟村	村庄	121.5985	42.06464
51		鞍山市千山风景名胜区温泉街道上石桥村	旅游景点	123.1113	41.01638
52		丹东市东港市北井子镇獐岛村	乡镇	123.8975	39.8682
53		抚顺市新宾满族自治县永陵镇赫图阿拉村	村庄	124.8596	41.702
54	吉林省	前郭尔罗斯蒙古族自治县查干湖渔场查干	旅游景点	124.4177	45.21005
55		延边朝鲜族自治州和龙市东城镇光东村	村庄	129.3399	42.76711
56		延边朝鲜族自治州和龙市西城镇金达莱村	NoClass	129.0687	42.68677
57		吉林市龙潭区乌拉街满族镇韩屯村	村庄	126.434	44.13164
58		吉林市舒兰市上营镇马鞍岭村	村庄	127.2521	44.14132
59		长白山保护开发区管理委员会池南区漫江村	旅游景点	127.5926	41.96338
60		长春市净月高新技术产业开发区玉潭镇友好	工业园区	125.4165	43.78017
61		辽源市东辽县安石镇朝阳村	村庄	125.3168	42.99301
62	黑龙江省	双鸭山市饶河县西林子乡小南河村	村庄	133.9242	46.93986
63		大兴安岭地区漠河市北极镇北红村	村庄	123.0666	53.50247
64		哈尔滨市宾县宾州镇友联村	村庄	127.3552	45.62838
65		牡丹江市宁安市渤海镇小朱家村	村庄	129.0901	44.06873
66		大庆市杜尔伯特蒙古族自治县连环湖镇南岗	村庄	124.1846	46.73015
67		七台河市勃利县青山乡奋斗村	村庄	130.6454	45.79773
68		伊春市新青区松林林场	购物	129.5977	48.20569
69		双鸭山市饶河县四排乡四排赫哲族村	乡镇	134.0541	46.96129
70		牡丹江市西安区海南乡中兴村	村庄	129.4195	44.53824
71		齐齐哈尔市铁锋区扎龙镇查罕诺村	村庄	124.0876	47.28107
72	上海市	金山区山阳镇渔业村	村庄	121.3718	30.73535
73		奉贤区青村镇吴房村	村庄	121.5309	30.89469
74		崇明区竖新镇仙桥村	村庄	121.605	31.63368
75		闵行区浦江镇革新村	村庄	121.5514	31.08022
76		崇明区竖新镇前卫村	乡镇	121.5996	31.61696
77		嘉定区马陆镇大裕村	村庄	121.3116	31.38313
78	江苏省	徐州市贾汪区潘安湖街道马庄村	村庄	117.345	34.36861
79		无锡市宜兴市湖㳇镇洑西村	村庄	119.7485	31.19951
80		南京市江宁区江宁街道黄龙岘茶文化村	旅游景点	118.6865	31.78207
81		常州市溧阳市戴埠镇李家园村	村庄	119.5263	31.2072
82		苏州市张家港市南丰镇永联村	村庄	120.7096	31.85132
83		淮安市洪泽区老子山镇龟山村	村庄	118.5377	33.12109
84		常州市金坛区薛埠镇仙姑村	村庄	119.3393	31.81581
85		无锡市锡山区东港镇山联村	村庄	120.5579	31.71084
86		南京市浦口区江浦街道不老村	村庄	118.6022	32.0935
87		苏州市常熟市支塘镇蒋巷村	地产小区	120.9629	31.50678
88		盐城市大丰区大中街道恒北村	村庄	120.4887	33.15356
89		南通市海门市常乐镇颐生村	村庄	121.2331	31.94427
90		泰州市泰兴市黄桥镇祁巷村	村庄	120.2799	32.21749
91	浙江省	湖州市长兴县水口乡顾渚村	村庄	119.8083	31.12677
92		湖州市安吉县天荒坪镇余村村	村庄	119.6096	30.52606
93		杭州市淳安县枫树岭镇下姜村	村庄	118.7203	29.37598

94		舟山市嵊泗县花鸟乡花鸟村	村庄	122.6909	30.84995
95		金华市兰溪市诸葛镇诸葛八卦村	旅游景点	119.2914	29.2505
96		衢州市开化县华埠镇金星村	村庄	118.3648	29.04794
97		丽水市龙泉市宝溪乡溪头村	村庄	118.7613	28.00821
98		宁波市宁海县前童镇鹿山村	村庄	121.3469	29.22744
99		嘉兴市秀洲区新塍镇潘家浜村	村庄	120.6067	30.75225
100		衢州市江山市大陈乡大陈村	村庄	118.5862	28.8219
101		台州市仙居县淡竹乡下叶村	村庄	120.5647	28.71077
102		宁波市奉化区萧王庙街道滕头村	村庄	121.3864	29.70248
103		丽水市遂昌县湖山乡红星坪村	村庄	118.9646	28.57537
104		温州市泰顺县竹里畲族乡竹里村	村庄	119.7673	27.70435
105	安徽省	黄山市黟县宏村镇宏村	旅游景点	117.985	30.00523
106		滁州市凤阳县小溪河镇小岗村	村庄	117.7697	32.83735
107		宣城市泾县桃花潭镇查济村	村庄	118.0319	30.51523
108		宿州市砀山县良梨镇良梨村	乡镇	116.5025	34.43461
109		黄山市徽州区西溪南镇西溪南村	村庄	118.2797	29.83863
110		合肥市巢湖市半汤街道汤山村	村庄	117.9145	31.66793
111		安庆市太湖县晋熙镇梅河村	村庄	116.2742	30.53931
112		滁州市天长市铜城镇龙岗村	乡镇	118.9341	32.88905
113		安庆市岳西县黄尾镇黄尾村	村庄	116.3183	31.1361
114		安庆市潜山市天柱山镇茶庄村	村庄	116.4686	30.7178
115		宣城市宁国市云梯畲族乡千秋村	村庄	119.2695	30.35924
116		宣城市广德县太极洞风景区桃园村	旅游景点	119.6024	31.11795
117	福建省	三明市泰宁县杉城镇际溪村	乡镇	117.1752	26.90425
118		龙岩市连城县宣和乡培田村	村庄	116.6326	25.64201
119		漳州市南靖县梅林镇官洋村	村庄	117.0899	24.66561
120		宁德市寿宁县下党乡下党村	村庄	119.3078	27.41095
121		平潭综合实验区流水镇北港村	NoClass	119.7059	25.48473
122		三明市尤溪县洋中镇桂峰村	村庄	118.5313	26.30595
123		宁德市寿宁县犀溪镇西浦村	村庄	119.6433	27.45491
124		漳州市长泰县马洋溪生态旅游区山重村	村庄	117.925	24.67294
125		泉州市惠安县崇武镇大岞村	村庄	118.9639	24.88492
126		宁德市福安市溪潭镇廉村村	村庄	119.6148	27.02507
127		南平市政和县石屯镇石圳村	村庄	118.7922	27.37731
128	江西省	上饶市婺源县江湾镇栗木坑村	村庄	118.1065	29.34609
129		赣州市大余县黄龙镇大龙村	村庄	114.3748	25.471
130		上饶市婺源县赋春镇源头村	旅游景点	117.6047	29.39902
131		吉安市井冈山市大陇镇大陇村	村庄	114.0266	26.63936
132		新余市仙女湖风景名胜区仰天岗办事处孝头	NoClass	114.7919	27.73228
133		宜春市靖安县中源乡三坪村	村庄	114.9619	28.8305
134		抚州市资溪县乌石镇新月村	村庄	116.9795	27.61533
135		赣州市龙南县临塘乡东坑村	乡镇	114.7996	24.78838
136		鹰潭市余江区杨溪乡琯溪村	乡镇	116.7468	28.23855
137		九江市永修县柘林镇易家河村	村庄	115.5249	29.19003
138		吉安市井冈山市厦坪镇菖蒲村	村庄	114.2642	26.70605
139		南昌市南昌县黄马乡凤凰村	乡镇	116.0123	28.34917
140	山东省	淄博市博山区池上镇中郝峪村	村庄	118.0425	36.30933

141		威海市荣成市宁津街道东楮岛村	旅游景点	122.5548	37.03855
142		临沂市沂南县铜井镇竹泉村	旅游景点	118.4329	35.64646
143		潍坊市青州市王府街道井塘村	村庄	118.3934	36.61865
144		临沂市沂水县院东头镇桃棵子村	村庄	118.3429	35.735
145		泰安市岱岳区道朗镇里峪村	村庄	116.9122	36.25224
146		济宁市邹城市石墙镇上九山村	旅游景点	116.8764	35.19351
147		济宁市梁山县大路口乡贾堌堆村	村庄	116.039	35.89089
148		临沂市兰陵县苍山街道压油沟村	村庄	117.9635	34.92882
149		日照市莒县东莞镇赵家石河村	村庄	118.9238	35.92982
150	河南省	洛阳市栾川县潭头镇重渡村	村庄	111.6929	33.93841
151		南阳市西峡县太平镇东坪村	村庄	111.7585	33.62051
152		焦作市温县赵堡镇陈家沟村	村庄	113.139	34.94537
153		三门峡市卢氏县官道口镇新坪村	乡镇	111.057	34.31243
154		开封市兰考县东坝头乡张庄村	村庄	114.8181	34.9262
155		信阳市新县八里畈镇丁李湾村	村庄	114.9491	31.73686
156		郑州市新郑市龙湖镇泰山村	NoClass	113.6373	34.5836
157		驻马店市平舆县东皇街道大王寨村	村庄	114.6595	32.99254
158		周口市淮阳县城关回族镇从庄村	村庄	114.9211	33.73997
159		鹤壁市淇县灵山街道赵庄村	村庄	114.097	35.66908
160	湖北省	荆州市石首市桃花山镇李花山村	村庄	112.7199	29.68311
161		黄冈市蕲春县檀林镇雾云山村	村庄	115.7448	30.64204
162		襄阳市保康县马桥镇尧治河村	村庄	110.8039	31.84432
163		十堰市竹山县文峰乡太和村	村庄	110.3615	32.24172
164		咸宁市通山县南林桥镇石门村	村庄	114.3605	29.63365
165		孝感市大悟县新城镇金岭村	村庄	114.3134	31.57864
166		荆门市钟祥市客店镇南庄村	村庄	112.8598	31.34353
167		恩施土家族苗族自治州利川市南坪乡营上[illegible]	村庄	108.8125	30.39347
168		恩施土家族苗族自治州恩施市白杨坪乡洞下[illegible]	村庄	109.6233	30.38397
169		宜昌市五峰县采花乡栗子坪村	村庄	110.567	30.16414
170		神农架林区宋洛乡盘龙村	乡镇	110.6032	31.66327
171	湖南省	西土家族苗族自治州花垣县双龙镇十八洞	村庄	109.5009	28.38818
172		郴州市汝城县文明瑶族乡沙洲瑶族村	村庄	113.3582	25.54694
173		湘潭市韶山市银田镇银田村	乡镇	112.6074	27.88399
174		益阳市南县乌嘴乡罗文村	村庄	112.446	29.35717
175		张家界市慈利县三官寺土家族乡罗潭村	村庄	110.6276	29.39623
176		长沙市长沙县果园镇浔龙河村	村庄	113.1819	28.35659
177		娄底市双峰县杏子铺镇双源村	村庄	112.2202	27.66762
178		常德市安乡县安康乡仙桃村	村庄	112.166	29.34722
179		永州市江永县兰溪瑶族乡勾蓝瑶村	餐饮	111.182	25.09988
180		衡阳市衡阳县西渡镇新桥村	村庄	112.3926	26.89426
181		岳阳市汨罗市白水镇西长村	村庄	112.9756	28.67192
182	广东省	梅州市大埔县西河镇北塘村	乡镇	116.747	24.48395
183		湛江市霞山区特呈岛村	NoClass	110.4315	21.15857
184		茂名市信宜市镇隆镇八坊村	村庄	110.8739	22.21359
185		河源市源城区埔前镇陂角村	村庄	114.6008	23.60889
186		惠州市龙门县南昆山生态旅游区中坪尾村	旅游景点	113.8802	23.64348
187		东莞市茶山镇南社村	村庄	113.8961	23.0678

188		佛山市顺德区杏坛镇逢简村	村庄	113.1435	22.81676
189		韶关市仁化县丹霞街道瑶塘新村	乡镇	113.749	25.08446
190		江门市台山市斗山镇浮石村	村庄	112.8531	22.05769
191		汕尾市陆河县水唇镇罗洞村	村庄	115.7697	23.2765
192	西壮族自治	贺州市富川瑶族自治县朝东镇岔山村	村庄	111.1512	25.05234
193		崇左市大新县堪圩乡明仕村	村庄	106.9166	22.70899
194		桂林市龙胜各族自治县龙脊镇大寨村	村庄	110.1517	25.80898
195		柳州市三江侗族自治县八江镇布央村	村庄	109.5119	25.85777
196		河池市巴马瑶族自治县那桃乡平林村	村庄	107.3288	24.1177
197		贵港市覃塘区覃塘街道龙凤村	村庄	109.4001	23.17239
198		南宁市马山县古零镇小都百屯	村庄	108.2478	23.67157
199		来宾市金秀瑶族自治县六巷乡大岭村	村庄	110.0693	23.94056
200		桂林市灵川县大圩镇袁家村	村庄	110.4529	25.19583
201		柳州市融水苗族自治县四荣乡荣地村	村庄	109.1229	25.32573
202		百色市田阳县五村镇巴某村	村庄	106.7516	23.52619
203	海南省	琼中黎族苗族自治县红毛镇什寒村	村庄	109.6463	19.0827
204		三亚市吉阳区中廖村	村庄	109.6264	18.33201
205		澄迈县老城镇罗驿村	村庄	110.1137	19.90008
206		白沙黎族自治县元门乡罗帅村	村庄	109.5464	19.09595
207		儋州市木棠镇铁匠村	村庄	109.3489	19.81811
208		海口市美兰区演丰镇山尾头村	村庄	110.5881	19.95714
209		定安县龙湖镇高林村	村庄	110.4806	19.58768
210		海口市秀英区永兴镇冯塘村	村庄	110.2424	19.80052
211	重庆市	永川区南大街街道黄瓜山村	NoClass	105.8386	29.24368
212		武隆区仙女山镇荆竹村	村庄	107.7416	29.38483
213		合川区涞滩镇二佛村	村庄	106.4799	30.17337
214		万盛经济技术开发区关坝镇凉风村	NoClass	106.8524	28.82785
215		大足区宝顶镇慈航社区	村庄	105.7561	29.74695
216		垫江县新民镇明月村	村庄	107.3924	30.40916
217		沙坪坝区曾家镇虎峰山村	村庄	106.2751	29.54681
218		荣昌区万灵镇大荣寨社区	村庄	105.6479	29.4903
219		巫溪县古路镇观峰村	村庄	109.3783	31.34216
220	四川省	成都市蒲江县甘溪镇明月村	村庄	103.3256	30.27826
221		德阳市绵竹市孝德镇年画村	旅游景点	104.2249	31.27925
222		成都市郫都区唐昌街道战旗村	村庄	103.7671	30.95642
223		凉山彝族自治州昭觉县支尔莫乡阿土列尔村	村庄	103.2678	28.05265
224		眉山市丹棱县顺龙乡幸福村	村庄	103.4146	30.05579
225		甘孜藏族自治州丹巴县聂呷乡甲居二村	村庄	101.8665	30.92942
226		成都市彭州市龙门山镇宝山村	村庄	103.7806	31.23409
227		乐山市峨边县黑竹沟镇底底古村	村庄	103.0841	29.02815
228		南充市阆中市天林乡五龙村	村庄	105.8548	31.47766
229		成都市都江堰市柳街镇七里社区	地产小区	103.6369	30.77813
230		泸州市纳溪区大渡口镇民强村	村庄	105.2207	28.72862
231		达州市宣汉县三墩土家族乡大窝村	村庄	108.3461	31.61767
232	贵州省	遵义市播州区枫香镇花茂村	村庄	106.5836	27.62383
233		铜仁市江口县太平镇云舍村	村庄	108.8165	27.74775
234		黔东南苗族侗族自治州台江县老屯乡长滩村	村庄	108.2848	26.7862

235		六盘水市盘州市淤泥乡岩博村	村庄	104.7347	25.99979
236		安顺市平坝区乐平镇塘约村	村庄	106.1625	26.44314
237		黔南布依族苗族自治州惠水县好花红镇好花纟	乡镇	106.656	26.13028
238		遵义市播州区平正仡佬族乡团结村	乡镇	106.5034	27.68563
239		遵义市新蒲新区新舟镇槐安村	村庄	107.1608	27.7971
240		贵阳市开阳县南江布依族苗族乡龙广村	村庄	106.9601	26.95434
241		黔西南布依族苗族自治州兴义市万峰林街道上纟	村庄	104.9192	24.99297
242		六盘水市水城县蟠龙镇百车河村	村庄	105.0745	26.44723
243		毕节市大方县核桃乡木寨村	村庄	105.6327	27.35097
244	云南省	大理白族自治州大理市双廊镇双廊村	村庄	100.1909	25.91849
245		大理白族自治州大理市双廊镇大建旁村	村庄	100.197	25.90563
246		文山壮族苗族自治州丘北县双龙营镇仙人洞	村庄	104.1271	24.12495
247		普洱市宁洱哈尼族彝族自治县同心镇那柯里	村庄	101.039	22.9139
248		昆明市安宁市温泉街道温泉小村	村庄	102.3663	24.99744
249		红河哈尼族彝族自治州建水县西庄镇团山村	村庄	102.7388	23.6551
250		昆明市宜良县耿家营乡河湾村	村庄	103.2465	25.05772
251		西双版纳傣族自治州勐海县打洛镇勐景来村	旅游景点	100.0835	21.69986
252		玉溪市红塔区大营街道大营街社区	道路	102.5192	24.32824
253		丽江市古城区大研街道义尚社区文林村民小	NoClass	100.2417	26.87839
254		西双版纳傣族自治州勐腊县勐腊镇曼龙勒村民	村庄	101.5846	21.53154
255		曲靖市罗平县鲁布革乡腊者村	乡镇	104.5371	24.77761
256		普洱市思茅区南屏镇曼连社区高家寨村民小	村庄	100.9816	22.75917
257	西藏自治区	拉萨市尼木县卡如乡卡如村	村庄	90.09217	29.35238
258		林芝市波密县古乡巴卡村	村庄	95.55016	29.87633
259		林芝市巴宜区林芝镇真巴村	村庄	94.45709	29.58317
260		昌都市江达县岗托镇岗托村	村庄	98.58671	31.6333
261		那曲市尼玛县文部乡南村	村庄	86.7615	31.34765
262		拉萨市当雄县羊八井镇巴嘎村	乡镇	90.53802	30.09832
263		拉萨市达孜区德庆镇白纳村	村庄	91.3922	29.64831
264		山南市隆子县玉麦乡玉麦村	乡镇	93.07251	28.63298
265		山南市错那县麻麻门巴民族乡麻麻村	乡镇	91.79683	27.87256
266	陕西省	咸阳市礼泉县烟霞镇袁家村	NoClass	108.5326	34.59251
267		商洛市商南县金丝峡镇太子坪村	村庄	110.5785	33.39917
268		商洛市柞水县营盘镇朱家湾村	村庄	108.9967	33.80802
269		榆林市佳县坑镇赤牛坬村	村庄	110.5718	37.84343
270		铜川市耀州区石柱镇马咀村	村庄	108.9113	34.98776
271		渭南市白水县杜康镇和家卓村	村庄	109.5285	35.21966
272		汉中市留坝县火烧店镇堰坎村	村庄	106.9051	33.53842
273		安康市石泉县饶峰镇胜利村	村庄	108.1642	33.1523
274		宝鸡市太白县黄柏塬镇黄柏塬村	村庄	107.514	33.81266
275		韩城市西庄镇党家村	旅游景点	110.4714	35.52861
276		安康市岚皋县四季镇天坪村	村庄	108.8613	32.3134
277	甘肃省	酒泉市敦煌市月牙泉镇月牙泉村	村庄	94.66583	40.09653
278		庆阳市华池县南梁镇荔园堡村	村庄	108.3377	36.39899
279		甘南藏族自治州卓尼县木耳镇博峪村	村庄	103.5448	34.55818
280		武威市天祝藏族自治县天堂镇天堂村	村庄	102.5094	36.94526
281		临夏回族自治州临夏市折桥镇折桥村	村庄	103.2522	35.63642

282		甘南藏族自治州碌曲县尕海乡尕秀村	村庄	102.2999	34.44297
283		酒泉市敦煌市阳关镇龙勒村	村庄	94.04278	39.94158
284		陇南市康县长坝镇花桥村	村庄	105.4146	33.43741
285		张掖市民乐县民联镇东寨村	村庄	100.8616	38.56467
286		甘南藏族自治州夏河县曲奥乡香告村	村庄	102.919	35.39448
287		庆阳市西峰区显胜乡毛寺村	村庄	107.634	35.44944
288		张掖市临泽县板桥镇红沟村	村庄	100.3498	39.19072
289	青海省	西宁市湟中县拦隆口镇拦一村	村庄	101.506	36.78125
290		海东市互助土族自治县东和乡麻吉村	村庄	102.0363	36.89564
291		西宁市湟中县土门关乡上山庄村	村庄	101.6774	36.44021
292		西宁市大通回族土族自治县朔北藏族乡边麻沟	村庄	101.8201	36.9685
293		西宁市湟中县田家寨镇田家寨村	村庄	101.7934	36.45133
294		海西蒙古族藏族自治州乌兰县茶卡镇莫河骆驼	NoClass	98.89778	36.84872
295		海东市互助土族自治县威远镇卓扎滩村	村庄	102.0094	36.8659
296	宁夏	西宁市湟源县日月藏族乡兔儿干村	村庄	101.1531	36.52201
297		中卫市沙坡头区迎水桥镇沙坡头村	村庄	105.0084	37.45321
298		固原市西吉县吉强镇龙王坝村	村庄	105.6303	35.96097
299		固原市隆德县陈靳乡新和村	村庄	106.1385	35.57546
300		银川市永宁县闽宁镇原隆村	村庄	105.9611	38.28305
301		固原市隆德县城关镇红崖村	村庄	106.1341	35.60596
302		石嘴山市大武口区长胜街道龙泉村	村庄	106.287	38.96052
303		吴忠市利通区上桥镇牛家坊村	村庄	106.2082	37.95553
304		银川市西夏区镇北堡镇镇北堡村	村庄	106.0679	38.62771
305		吴忠市盐池县高沙窝镇兴武营村	村庄	107.0392	38.10782
306	新疆	乌鲁木齐市乌鲁木齐县水西沟镇平西梁村	村庄	87.44935	43.45694
307		阿勒泰地区布尔津县禾木喀纳斯蒙古族乡禾木	购物	87.43053	48.57018
308		伊犁哈萨克自治州特克斯县喀拉达拉镇琼库什	村庄	82.19575	42.91965
309		吐鲁番市高昌区亚尔镇上湖村	村庄	89.105	42.97927
310		塔城地区额敏县加尔布拉克农场酒花村	村庄	83.69113	46.59427
311		巴音郭楞蒙古自治州库尔勒市巴州阿瓦提农	NoClass	86.11181	41.66159
312		克拉玛依市乌尔禾区乌尔禾镇哈克村	村庄	85.65932	46.11614
313		哈密市巴里坤哈萨克自治县石人子乡石人子	村庄	93.24309	43.59494
314		阿克苏地区阿瓦提县英艾日克镇恰其村	村庄	80.16228	40.88656
315		第四师可克达拉市62团金边镇	村庄	80.99368	43.93612
316		第十师北屯市185团3连	村庄	87.80976	47.32763
317		第二师铁门关市31团2连	区县	85.67798	41.85613
318		第一师阿拉尔市11团13连	村庄	79.52275	41.40472
319		第八师石河子市152团10连	NoClass	86.05147	44.20618
320		第四师可克达拉市78团5连	村庄	81.00024	43.9477
