# Supplementary material for: The spatial distribution characteristics and influencing factors of key villages in rural tourism in China
Source: PLoS One. 2025 Aug 19;20(8):e0330486. doi: 10.1371/journal.pone.0330486 (PMC12364373; doi:10.1371/journal.pone.0330486)
Supplement: S2 File — (PDF) [file pone.0330486.s002.pdf]

ID				经度	纬度
1		北京市密云区古北口镇司马台村	村庄	117.2387	40.65466
2		北京市延庆区旧县镇盆窑村	村庄	116.0877	40.53714
3		北京市平谷区金海湖镇将军关村	村庄	117.3136	40.27009
4		北京市房山区大石窝镇王家磨村	村庄	115.722	39.55541
5		北京市门头沟区清水镇洪水口村	村庄	115.4783	39.99238
6		北京市怀柔区汤河口镇庄户沟门村	村庄	116.6239	40.73344
7		天津市蓟州区穿芳峪镇东水厂村	村庄	117.5193	40.14122
8		天津市蓟州区下营镇前干涧村	村庄	117.416	40.2267
9		天津市津南区北闸口镇前进村	村庄	117.3797	38.91533
10		天津市北辰区青光镇韩家墅村	村庄	117.0799	39.19224
11		天津市宁河区板桥镇盆罐庄村	村庄	117.8217	39.48389
12		河北省邯郸市武安市淑村镇白沙村	村庄	114.22	36.57646
13		河北省张家口市张北县小二台镇德胜村	村庄	114.8343	41.18231
14		河北省石家庄市平山县西柏坡镇北庄村	村庄	113.9136	38.3344
15		河北省衡水市故城县房庄镇吴梧茂村	村庄	115.9444	37.46056
16		河北省邢台市内丘县侯家庄乡岗底村	村庄	114.0905	37.32485
17		河北省承德市隆化县七家镇温泉村	村庄	118.1054	41.48172
18		河北省邢台市宁晋县贾家口镇黄儿营西村	村庄	115.1095	37.60004
19		山西省太原市娄烦县静游镇峰岭底村	村庄	111.858	38.16255
20		山西省忻州市偏关县老牛湾镇老牛湾村	村庄	111.4356	39.63699
21		山西省阳泉市盂县孙家庄镇王炭咀村	村庄	113.4818	38.12408
22		山西省晋中市寿阳县宗艾镇下洲村	村庄	113.1243	37.98933
23		山西省长治市平顺县石城镇岳家寨村	村庄	113.6345	36.29993
24		山西省临汾市永和县乾坤湾乡东征村	村庄	110.4941	36.67602
25		山西省运城市河津市清涧街道龙门村	村庄	110.6357	35.66228
26		内蒙古自治区赤峰市松山区大庙镇小庙子村	村庄	118.1414	42.37248
27		内蒙古自治区呼和浩特市和林格尔县新店子镇胶泥湾村	村庄	111.999	40.23609
28		内蒙古自治区兴安盟阿尔山市明水河镇西口村	村庄	120.6961	46.70935
29		内蒙古自治区呼伦贝尔市鄂温克族自治旗巴彦塔拉达斡尔民族乡伊[illegible]	乡镇	119.7375	49.00307
30		内蒙古自治区乌兰察布市兴和县店子镇卢家营村	村庄	113.8486	40.62386
31		内蒙古自治区巴彦淖尔市杭锦后旗双庙镇太荣村	村庄	106.7642	40.7979
32		辽宁省锦州市凌海市温滴楼镇边墙子村	村庄	121.0691	41.2045
33		辽宁省朝阳市凌源市大王杖子乡宫家烧锅村	村庄	119.2989	41.20738
34		辽宁省辽阳市辽阳县刘二堡镇前杜村	村庄	122.8231	41.19445
35		辽宁省营口市鲅鱼圈区芦屯镇小望海村	村庄	122.1872	40.31236
36		辽宁省葫芦岛市兴城市三道沟满族乡头道沟村	村庄	120.1799	40.58558
37		吉林省长春市双阳区太平镇小石村	村庄	125.7546	43.37485
38		吉林省吉林市桦甸市桦郊乡晓光村	村庄	126.7404	42.91264
39		吉林省延边朝鲜族自治州龙井市智新镇明东村	村庄	129.5394	42.67217
40		吉林省白山市长白朝鲜族自治县马鹿沟镇果园民俗村	休闲娱乐	127.9354	41.45496
41		吉林省通化市东昌区金厂镇夹皮沟村	村庄	125.8743	41.61625
42		吉林省白城市通榆县向海蒙古族乡向海村	村庄	122.3388	45.03001
43		黑龙江省齐齐哈尔市梅里斯达斡尔族区雅尔塞镇哈拉新村	村庄	123.9038	47.45906
44		黑龙江省牡丹江市穆棱市下城子镇孤榆树村	村庄	130.4407	44.60832
45		黑龙江省佳木斯市同江市八岔赫哲族乡八岔村	村庄	133.8898	48.21135
46		黑龙江省大庆市林甸县四合乡联合村	村庄	124.9131	47.29746

47		黑龙江省黑河市爱辉区瑷珲镇外三道沟村	村庄	127.4892	50.06268
48		黑龙江省伊春市铁力市工农乡北星村	村庄	128.1945	47.07631
49		上海市青浦区金泽镇莲湖村	村庄	120.9982	31.06125
50		上海市金山区漕泾镇水库村	村庄	121.3919	30.82647
51		上海市嘉定区安亭镇向阳村	村庄	121.1329	31.3062
52		上海市宝山区月浦镇聚源桥村	村庄	121.381	31.44139
53		上海市崇明区新河镇井亭村	村庄	121.4881	31.59007
54		江苏省南京市浦口区永宁街道大埝社区	NoClass	118.6119	32.12961
55		江苏省苏州市吴中区越溪街道旺山村	村庄	120.5479	31.21027
56		江苏省徐州市沛县大屯街道安庄社区	村庄	116.8491	34.87741
57		江苏省盐城市东台市弶港镇巴斗村	村庄	120.8742	32.87239
58		江苏省扬州市邗江区方巷镇沿湖村	村庄	119.4268	32.58558
59		江苏省泰州市高港区白马镇陈家村	村庄	119.973	32.39239
60		江苏省宿迁市宿城区耿车镇刘圩村	乡镇	118.1821	33.91376
61		浙江省杭州市余杭区径山镇小古城村	村庄	119.8667	30.39274
62		浙江省湖州市吴兴区妙西镇妙山村	村庄	119.9836	30.76956
63		浙江省绍兴市柯桥区湖塘街道香林村	村庄	120.4142	30.03088
64		浙江省金华市武义县俞源乡俞源村	乡镇	119.6595	28.77579
65		浙江省舟山市定海区马岙街道马岙村	村庄	122.0893	30.11855
66		浙江省台州市天台县街头镇后岸村	村庄	120.8128	29.08618
67		浙江省丽水市云和县赤石乡赤石村	村庄	119.442	28.13459
68		安徽省黄山市徽州区潜口镇唐模村	旅游景点	118.3314	29.8706
69		安徽省滁州市南谯区施集镇井楠村	村庄	118.1078	32.26618
70		安徽省芜湖市湾沚区六郎镇官巷村	道路	118.527	31.27688
71		安徽省池州市青阳县朱备镇将军村	村庄	117.8803	30.49411
72		安徽省安庆市岳西县河图镇南河村	村庄	116.0749	30.84164
73		安徽省淮北市杜集区矿山集街道南山村	村庄	116.9235	33.97
74		安徽省蚌埠市怀远县龙亢镇龙亢村	乡镇	116.8863	33.13865
75		福建省泉州市晋江市新塘街道梧林社区	村庄	118.593	24.75657
76		福建省宁德市福鼎市磻溪镇赤溪村	旅游景点	120.1064	27.0685
77		福建省漳州市华安县高安镇坪水村	村庄	117.3617	24.84999
78		福建省三明市沙县区夏茂镇俞邦村	村庄	117.6501	26.59075
79		福建省龙岩市永定区湖坑镇南江村	村庄	116.9829	24.58763
80		福建省平潭综合实验区君山镇磹水村	NoClass	119.7059	25.48473
81		江西省赣州市于都县梓山镇潭头村	村庄	115.4923	25.95603
82		江西省上饶市婺源县思口镇思溪村延村	旅游景点	117.7912	29.34052
83		江西省赣州市瑞金市叶坪镇华屋村	购物	116.1506	25.89364
84		江西省九江市庐山市白鹿镇秀峰村	村庄	115.9934	29.46725
85		江西省景德镇市浮梁县瑶里镇五华村	乡镇	117.5786	29.56113
86		江西省抚州市黎川县德胜镇德胜村	村庄	117.0079	27.17465
87		江西省南昌市新建区溪霞镇店前村	村庄	115.8194	28.88999
88		山东省济南市莱芜区雪野街道房干村	村庄	117.4466	36.43349
89		山东省日照市岚山区岚山头街道官草汪村	村庄	119.3569	35.10175
90		山东省济宁市曲阜市尼山镇鲁源村	村庄	117.184	35.49513
91		山东省烟台市蓬莱区大辛店镇木兰沟村	村庄	120.8897	37.60437
92		山东省淄博市高青县常家镇蓑衣樊村	村庄	117.8847	37.23498
93		山东省青岛市崂山区王哥庄街道晓望社区	地产小区	120.6456	36.25567

94		山东省潍坊市临朐县嵩山生态旅游发展服务中心淹子岭村	村庄	118.2369	36.39239
95		河南省郑州市巩义市小关镇南岭新村	村庄	113.131	34.65906
96		河南省郑州市巩义市大峪沟镇海上桥村	村庄	113.0683	34.74475
97		河南省洛阳市嵩县车村镇天桥沟村	村庄	111.9411	33.762
98		河南省焦作市修武县西村乡大南坡村	村庄	113.3176	35.34608
99		河南省濮阳市清丰县双庙乡单拐村	村庄	115.2133	35.82567
100		河南省信阳市浉河区浉河港镇郝家冲村	村庄	113.9065	32.02063
101		河南省周口市西华县红花集镇龙池头村	村庄	114.4647	33.81398
102		湖北省襄阳市襄城区尹集乡姚庵村	村庄	112.0575	31.94958
103		湖北省宜昌市远安县花林寺镇龙凤村	村庄	111.5875	30.92376
104		湖北省荆州市松滋市洈水镇樟木溪村	村庄	111.5333	30.00546
105		湖北省孝感市孝昌县小悟乡田堂村	村庄	114.1016	31.30988
106		湖北省黄冈市罗田县骆驼坳镇燕窝垸村	村庄	115.411	30.68861
107		湖北省咸宁市通城县大坪乡内冲瑶族村	村庄	113.7049	29.37089
108		湖北省随州市广水市武胜关镇桃源村	乡镇	114.0576	31.59995
109		湖南省湘西土家族苗族自治州凤凰县麻冲乡竹山村	村庄	109.4298	27.98705
110		湖南省怀化市溆浦县统溪河镇穿岩山村	乡镇	110.5597	27.74903
111		湖南省邵阳市洞口县罗溪瑶族乡宝瑶村	村庄	110.2296	27.1071
112		湖南省娄底市新化县吉庆镇油溪桥村	村庄	111.4856	28.00822
113		湖南省衡阳市珠晖区茶山坳镇堰头村	村庄	112.7013	26.96857
114		湖南省常德市津市市金鱼岭街道大关山村	村庄	111.8293	29.59832
115		湖南省张家界市永定区王家坪镇马头溪村	村庄	110.8004	29.01421
116		广东省广州市从化区城郊街西和村	村庄	113.5833	23.62613
117		广东省肇庆市封开县江口街道台洞村	村庄	111.5141	23.5013
118		广东省惠州市惠阳区秋长街道周田村	村庄	114.419	22.8587
119		广东省汕头市潮南区陇田镇东华村	村庄	116.5273	23.1749
120		广东省中山市南朗街道左步村	村庄	113.5468	22.50545
121		广东省湛江市徐闻县角尾乡放坡村	村庄	109.9259	20.25978
122		广东省潮州市潮安区凤凰镇叫水坑村	村庄	116.7394	23.85395
123		广西壮族自治区柳州市融水苗族自治县香粉乡雨卜村	村庄	109.1954	25.31424
124		广西壮族自治区桂林市兴安县华江瑶族乡龙塘江村	村庄	110.438	25.80311
125		广西壮族自治区钦州市浦北县北通镇那新村	村庄	109.3548	22.214
126		广西壮族自治区百色市凌云县伶站瑶族乡浩坤村	乡镇	106.5952	24.16628
127		广西壮族自治区贺州市昭平县黄姚镇北莱村	村庄	111.2637	24.29755
128		广西壮族自治区河池市南丹县里湖瑶族乡朵努社区	乡镇	107.6601	25.11134
129		广西壮族自治区来宾市金秀瑶族自治县金秀镇六段村	村庄	110.2024	24.23621
130		海南省琼海市博鳌镇留客村	村庄	110.5317	19.13505
131		海南省万宁市兴隆华侨农场57队	NoClass	110.2491	18.69251
132		海南省儋州市中和镇七里村	村庄	109.3675	19.75826
133		海南省昌江黎族自治县王下乡浪论村	村庄	109.1759	18.99521
134		海南省文昌市潭牛镇大庙村	村庄	110.7121	19.71635
135		重庆市万州区长岭镇安溪村	村庄	108.5053	30.79323
136		重庆市九龙坡区铜罐驿镇英雄湾村	村庄	106.3677	29.32259
137		重庆市江津区先锋镇保坪村	村庄	106.2436	29.18415
138		重庆市巫山县竹贤乡下庄村	村庄	110.0218	31.28154
139		重庆市奉节县兴隆镇回龙村	村庄	109.5121	30.79839
140		重庆市潼南区崇龛镇明月社区	乡镇	105.6147	30.1565

141		四川省成都市邛崃市平乐镇花楸村	村庄	103.2836	30.39113
142		四川省乐山市金口河区永和镇胜利村	村庄	103.0203	29.29081
143		四川省宜宾市翠屏区李庄镇高桥村	村庄	104.7716	28.76272
144		四川省广安市岳池县白庙镇郑家村	村庄	106.3693	30.56505
145		四川省雅安市石棉县安顺场镇安顺村	村庄	102.2812	29.27579
146		四川省眉山市青神县青竹街道兰沟村	村庄	103.822	29.81614
147		四川省阿坝藏族羌族自治州小金县四姑娘山镇长坪村	村庄	102.844	31.00162
148		贵州省毕节市黔西市新仁苗族乡化屋村	村庄	106.1146	26.80882
149		贵州省六盘水市水城区米箩镇倮么村	乡镇	105.0014	26.38231
150		贵州省安顺市平坝区夏云镇小河湾村	旅游景点	106.3296	26.41425
151		贵州省贵阳市开阳县禾丰布依族苗族乡马头村	村庄	106.9134	26.92876
152		贵州省遵义市湄潭县鱼泉街道新石社区	村庄	107.4445	27.80263
153		贵州省黔东南苗族侗族自治州锦屏县敦寨镇雷屯村	村庄	109.2442	26.44094
154		贵州省黔南布依族苗族自治州龙里县龙山镇龙山社区	乡镇	106.9834	26.45808
155		云南省大理白族自治州鹤庆县草海镇新华村	村庄	100.1759	26.62228
156		云南省丽江市玉龙纳西族自治县拉市镇均良村	村庄	100.1309	26.90315
157		云南省玉溪市澄江市龙街镇禄充社区	餐饮	102.8408	24.56154
158		云南省临沧市双江拉祜族佤族布朗族傣族自治县沙河乡允俸村	村庄	99.81006	23.47737
159		云南省西双版纳傣族自治州勐海县打洛镇曼掌村	村庄	100.0405	21.68214
160		云南省曲靖市宣威市东山镇芙蓉村	村庄	104.1857	26.15467
161		云南省文山壮族苗族自治州丘北县双龙营镇普者黑村	村庄	104.1186	24.13947
162		西藏自治区日喀则市定结县琼孜乡姆村	乡镇	88.00849	28.21463
163		西藏自治区日喀则市亚东县帕里镇四居委会	NoClass	89.14606	27.77572
164		西藏自治区山南市洛扎县色乡色村	乡镇	90.82225	28.1853
165		西藏自治区山南市洛扎县拉郊乡拉郊村	村庄	91.00607	28.08889
166		西藏自治区林芝市米林县南伊珞巴民族乡南伊村	购物	94.1999	29.17232
167		陕西省西安市鄠邑区石井街道蔡家坡村	村庄	108.632	34.02253
168		陕西省宝鸡市金台区金河镇周家庄村	村庄	107.1036	34.47433
169		陕西省渭南市合阳县黑池镇南社社区	村庄	110.2065	35.07334
170		陕西省汉中市南郑区汉山街道汉山村	村庄	106.9338	32.94895
171		陕西省商洛市山阳县法官镇法官庙村	村庄	110.0921	33.25845
172		陕西省延安市宝塔区万花山镇佛道坪村	村庄	109.3153	36.51028
173		甘肃省兰州市榆中县小康营乡浪街村	村庄	104.1396	35.80273
174		甘肃省定西市渭源县田家河乡元古堆村	村庄	103.9348	35.04309
175		甘肃省武威市凉州区高坝镇蜻蜓村	村庄	102.7209	37.91297
176		甘肃省白银市白银区水川镇顾家善村	村庄	104.21	36.34029
177		甘肃省临夏回族自治州康乐县八松乡纳沟村	村庄	103.4824	35.26514
178		甘肃省庆阳市庆城县庆城镇药王洞村	村庄	107.8932	35.99659
179		青海省西宁市大通回族土族自治县朔北藏族乡东至沟村	村庄	101.8131	36.97905
180		青海省海东市化隆回族自治县群科镇安达其哈村	村庄	102.0047	36.00822
181		青海省黄南藏族自治州同仁市扎毛乡扎毛村	村庄	101.8844	35.34792
182		青海省海东市乐都区高庙镇新庄村	村庄	102.5442	36.46512
183		青海省海南藏族自治州贵德县河西镇团结村	村庄	101.4043	36.03585
184		宁夏回族自治区吴忠市青铜峡市大坝镇韦桥村	村庄	105.9954	37.95075
185		宁夏回族自治区吴忠市红寺堡区红寺堡镇弘德村	村庄	106.1119	37.48339
186		宁夏回族自治区石嘴山市平罗县高仁乡六顷地村	村庄	106.6425	38.76196
187		宁夏回族自治区中卫市中宁县余丁乡黄羊村	村庄	105.5348	37.50977

188		宁夏回族自治区固原市隆德县凤岭乡李士村	村庄	105.967	35.55343
189		新疆维吾尔自治区阿勒泰地区喀纳斯景区铁热克提乡白哈巴村	NoClass	86.69907	48.47497
190		新疆维吾尔自治区阿勒泰地区吉木乃县托斯特乡塔斯特村（石头村	村庄	86.07075	47.27429
191		新疆维吾尔自治区伊犁哈萨克自治州新源县那拉提镇拜依盖托别	乡镇	84.00105	43.32699
192		新疆维吾尔自治区昌吉回族自治州昌吉市六工镇十三户村	村庄	87.45248	44.12828
193		新疆维吾尔自治区克拉玛依市乌尔禾区乌尔禾镇查干草村	村庄	85.67737	46.11433
194		新疆维吾尔自治区吐鲁番市高昌区葡萄镇巴格日社区	乡镇	89.20303	42.95366
195		新疆生产建设兵团第六师红旗农场11连	政府机构	89.11583	44.24669
