# Supplementary material for: The spatial distribution characteristics and influencing factors of key villages in rural tourism in China
Source: PLoS One. 2025 Aug 19;20(8):e0330486. doi: 10.1371/journal.pone.0330486 (PMC12364373; doi:10.1371/journal.pone.0330486)
Supplement: S3 File — (PDF) [file pone.0330486.s003.pdf]

ID				经度	纬度
1	北京	门头沟区斋堂镇爨底下村	村庄	115.64	39.99459
2	北京	延庆区刘斌堡乡小观头村	村庄	116.2366	40.56055
3	北京	延庆区八达岭镇石峡村	村庄	115.9482	40.30399
4	北京	怀柔区渤海镇六渡河村	村庄	116.5311	40.38334
5	北京	密云区溪翁庄镇金叵罗村	村庄	116.8512	40.45485
6	北京	顺义区龙湾屯镇焦庄户村	村庄	116.866	40.23293
7	北京	延庆区旧县镇东龙湾村	村庄	116.0925	40.51322
8	北京	怀柔区琉璃庙镇双文铺村	村庄	116.6751	40.65252
9	北京	怀柔区九渡河镇西水峪村	村庄	116.3072	40.40753
10	北京	怀柔区怀柔镇芦庄村	村庄	116.6151	40.37032
11	北京	房山区十渡镇平峪村	村庄	115.5399	39.65819
12	北京	顺义区马坡镇石家营村	村庄	116.6084	40.1898
13	北京	平谷区镇罗营镇玻璃台村	村庄	117.2241	40.32623
14	北京	延庆区张山营镇后黑龙庙村	村庄	115.8409	40.45506
15	北京	房山区张坊镇穆家口村	村庄	115.6624	39.62647
16	北京	怀柔区琉璃庙镇白河北村	村庄	116.676	40.66281
17	北京	平谷区山东庄镇鱼子山村	村庄	117.1638	40.22384
18	北京	昌平区十三陵镇仙人洞村	村庄	116.2382	40.24922
19	北京	怀柔区雁栖镇官地村	村庄	116.6292	40.42744
20	北京	平谷区镇罗营镇张家台村	村庄	117.2148	40.32811
21	北京	密云区巨各庄镇蔡家洼村	村庄	116.889	40.37346
22	北京	平谷区金海湖镇黄草洼村	村庄	117.3296	40.18826
23	北京	延庆区井庄镇三司村	村庄	116.1188	40.46061
24	天津	西青区辛口镇水高庄村	村庄	116.9344	39.07515
25	天津	北辰区西堤头镇赵庄子村	村庄	117.3271	39.29446
26	天津	宝坻区黄庄镇小辛码头村	村庄	117.4517	39.52242
27	天津	蓟州区下营镇东山村	村庄	117.5049	40.18526
28	天津	蓟州区官庄镇砖瓦窑村	村庄	117.2839	40.08807
29	天津	西青区辛口镇大杜庄村	村庄	116.9727	39.04783
30	天津	蓟州区穿芳峪镇英歌寨村	村庄	117.5373	40.1057
31	天津	蓟州区官庄镇联合村	村庄	117.2972	40.08177
32	天津	宝坻区牛家牌镇赵家湾村	村庄	117.3694	39.51486
33	天津	蓟州区下营镇青山岭村	村庄	117.4927	40.19601
34	天津	西青区辛口镇第六埠村	村庄	116.9241	39.06589
35	河北	承德市滦平县巴克什营镇花楼沟村	村庄	117.2426	40.69671
36	河北	保定市阜平县龙泉关镇骆驼湾村	村庄	113.8448	38.89018
37	河北	承德市围场县御道口乡御道口村	村庄	116.9728	42.14324
38	河北	唐山市迁安市大五里乡山叶口村	旅游景点	118.5212	39.98305
39	河北	保定市易县安格庄乡安格庄村	村庄	115.2353	39.28406
40	河北	邢台市内丘县南赛乡神头村	村庄	114.2681	37.30801
41	河北	邯郸市涉县井店镇刘家村	村庄	113.8516	36.5573
42	河北	廊坊市香河县蒋辛屯镇北李庄村	村庄	116.9528	39.84486
43	河北	石家庄市灵寿县南营乡车谷砣村	汽车服务	113.9378	38.61055
44	河北	秦皇岛市北戴河区戴河镇西古城村	村庄	119.4152	39.81332
45	河北	唐山市迁安市大崔庄镇白羊峪村	村庄	118.7219	40.19387
46	河北	邢台市信都区浆水镇前南峪村	村庄	113.9338	37.16591

47	河北	石家庄市井陉县南障城镇吕家村	村庄	113.9963	37.90027
48	河北	石家庄市晋州市周家庄乡第九生产队	乡镇	115.0921	38.02203
49	河北	承德市丰宁县大滩镇小北沟村	村庄	116.1442	41.57683
50	河北	沧州市青县曹寺乡张广王村	村庄	116.7523	38.55677
51	河北	邯郸市邯山区河沙镇镇小堤村	村庄	114.5632	36.50492
52	河北	秦皇岛市北戴河区海滨镇陆庄村	村庄	119.4338	39.81032
53	河北	保定市阜平县龙泉关镇顾家台村	村庄	113.8529	38.91117
54	河北	邢台市信都区路罗镇英谈村	村庄	113.8424	37.10993
55	河北	邯郸市峰峰矿区和村镇东和村	村庄	114.1153	36.53041
56	河北	秦皇岛市青龙满族自治县隔河头乡花果山村	村庄	119.3509	40.12769
57	河北	保定市易县西陵镇凤凰台村	村庄	115.3633	39.36331
58	河北	邯郸市涉县关防乡后池村	村庄	113.9092	36.53749
59	山西	朔州市怀仁市马辛庄乡鲁沟村	村庄	113.2946	39.87413
60	山西	太原市阳曲县黄寨镇上安村	村庄	112.657	38.10708
61	山西	长治市武乡县蟠龙镇砖壁村	村庄	113.2156	36.75572
62	山西	长治市壶关县桥上乡大河村	村庄	113.6096	35.93137
63	山西	临汾市安泽县府城镇飞岭村	村庄	112.2661	36.20789
64	山西	晋城市陵川县附城镇丈河村	村庄	113.1997	35.59271
65	山西	忻州市忻府区合索乡北合索村	村庄	112.5812	38.46186
66	山西	阳泉市城区义井镇小河村	村庄	113.6415	37.84118
67	山西	晋中市介休市龙凤镇南庄村	村庄	111.9577	36.98241
68	山西	晋城市城区钟家庄街道洞头村	旅游景点	112.8839	35.43394
69	山西	晋中市榆次区乌金山镇后沟村	村庄	112.7995	37.83527
70	山西	阳泉市郊区平坦镇桃林沟村	旅游景点	113.5502	37.89362
71	山西	晋城市泽州县金村镇东六庄村	村庄	112.9402	35.51199
72	山西	大同市灵丘县红石塄乡下车河村	村庄	114.281	39.36292
73	山西	运城市永济市开张镇东开张村	村庄	110.5581	34.94914
74	山西	临汾市曲沃县里村镇朝阳村	村庄	111.4543	35.74229
75	山西	太原市娄烦县天池店乡河北村	村庄	111.875	37.93336
76	山西	晋城市阳城县润城镇中庄村	村庄	112.5491	35.51515
77	内蒙古	贝尔市额尔古纳市恩和俄罗斯族民族乡恩	区县	120.1728	50.24095
78	内蒙古	锡林郭勒盟太仆寺旗宝昌镇边墙村	村庄	115.3747	41.87957
79	内蒙古	乌海市海南区西卓子山街道赛汗乌素村	村庄	106.7748	39.38471
80	内蒙古	通辽市经济技术开发区河西街道湛路村	村庄	122.1595	43.59315
81	内蒙古	呼和浩特市回民区攸攸板镇东乌素图村	村庄	111.5708	40.84282
82	内蒙古	锡林郭勒盟多伦县滦源镇大孤山村	村庄	116.7356	42.18106
83	内蒙古	乌兰察布市凉城县岱海镇三苏木村	乡镇	112.5009	40.53829
84	内蒙古	尔多斯市伊金霍洛旗伊金霍洛镇布拉格嘎	村庄	109.8002	39.34496
85	内蒙古	呼和浩特市赛罕区黄河少镇石人湾村	村庄	112.0733	40.76167
86	内蒙古	兴安盟阿尔山白狼镇林俗村	休闲娱乐	120.0989	47.03844
87	内蒙古	巴彦淖尔市五原县塔尔湖镇联丰村	村庄	108.0067	40.94441
88	内蒙古	赤峰市喀喇沁旗十家乡林营子村	村庄	118.891	41.94096
89	内蒙古	通辽市奈曼旗白音他拉苏木庙屯村	乡镇	120.7861	43.06663
90	内蒙古	和浩特市托克托县河口管理委员会郝家窑	政府机构	111.1483	40.27031
91	内蒙古	阿拉善盟阿右旗巴丹吉林镇额肯呼都格嘎	村庄	101.6776	39.19618
92	辽宁	辽阳市弓长岭区汤河镇柳河汤村	村庄	123.3663	41.12565
93	辽宁	本溪市桓仁满族自治县向阳乡和平村	村庄	125.3177	41.13224

94	辽宁	丹东市东港市孤山镇大鹿岛村	乡镇	123.5898	39.89758
95	辽宁	溪市桓仁满族自治县雅河朝鲜族乡湾湾川	村庄	125.2702	41.1996
96	辽宁	本溪市南芬区思山岭街道解放村	村庄	123.7149	41.14479
97	辽宁	鞍山市千山风景名胜区韩家峪村	村庄	123.1651	41.02125
98	辽宁	营口市盖州市双台镇思拉堡村	村庄	122.2505	40.23731
99	辽宁	大连市庄河市步云山乡步云山村	村庄	122.7576	40.071
100	辽宁	大连市金普新区石河街道石河村	村庄	121.8548	39.33373
101	辽宁	阜新市阜新蒙古族自治县佛寺镇佛寺村	村庄	121.4435	41.93655
102	辽宁	沈阳市法库县大孤家子镇半拉山子村	村庄	123.303	42.33608
103	辽宁	盘锦市大洼区荣兴街道荣兴村	村庄	122.0882	40.77526
104	辽宁	锦州市义县瓦子峪镇大铁厂村	村庄	121.5269	41.62736
105	辽宁	本溪市本溪满族自治县草河掌镇胡堡村	乡镇	124.0427	41.0679
106	辽宁	鞍山市千山区东鞍山街道对桩石村	村庄	123.05	40.99921
107	辽宁	本溪市明山区卧龙街道韩家村	村庄	123.9219	41.23556
108	辽宁	朝阳市北票市大黑山特别管理区西苍村	村庄	120.5091	42.00338
109	辽宁	大连市庄河市仙人洞镇马道口村	村庄	122.9746	40.06508
110	辽宁	市喀喇沁左翼蒙古族自治县平房子镇小	村庄	119.6535	41.04583
111	辽宁	本溪市桓仁满族自治县普乐堡镇老漫子	村庄	125.0514	41.1511
112	辽宁	铁岭市银州区龙山乡七里屯村	村庄	123.8844	42.27779
113	吉林	延边朝鲜族自治州珲春市敬信镇防川村	村庄	130.5865	42.43745
114	吉林	春市莲花山生态旅游度假区泉眼镇泉眼	旅游景点	125.54	43.88373
115	吉林	吉林市丰满区江南乡孟家村	村庄	126.675	43.75582
116	吉林	长春市九台区土门岭街道马鞍山村	村庄	126.0268	44.11665
117	吉林	通化市柳河县安口镇青沟子村	村庄	125.6218	42.17349
118	吉林	通化市集安市太王镇钱湾村	乡镇	126.2049	41.13572
119	吉林	延边朝鲜族自治州安图县万宝镇红旗村	村庄	128.298	42.85327
120	吉林	延边朝鲜族自治州汪清县大兴沟镇红日	村庄	129.4569	43.47223
121	吉林	长春市农安县华家镇战家村	村庄	125.0219	44.27208
122	吉林	四平市伊通满族自治县河源镇保南村	村庄	125.736	43.10809
123	吉林	延边朝鲜族自治州敦化市雁鸣湖镇大山	村庄	128.5926	43.68567
124	吉林	延边朝鲜族自治州敦化市雁鸣湖镇小山	乡镇	128.5969	43.687
125	吉林	通化市辉南县金川镇金川村	乡镇	126.3893	42.34747
126	吉林	延边朝鲜族自治州图们市石岘镇水南村	村庄	129.8137	43.02893
127	吉林	吉林市蛟河市漂河镇富江村	村庄	127.2376	43.53511
128	吉林	通化市通化县西江镇岔信村	村庄	125.6858	41.56005
129	吉林	白山市临江市四道沟镇坡口村	村庄	127.0664	41.73254
130	吉林	吉林市永吉县北大湖镇草庙子村	村庄	126.4741	43.40487
131	吉林	通化市东昌区金厂镇上龙头村	村庄	126.0032	41.61056
132	黑龙江	黑河市爱辉区瑷珲镇外四道沟村	村庄	127.5033	50.08061
133	黑龙江	伊春市上甘岭林业局溪水林场	政府机构	129.0168	47.9723
134	黑龙江	哈尔滨市尚志市鱼池乡新兴村	村庄	128.668	44.90825
135	黑龙江	鸡西市虎林市虎头镇虎头村	村庄	133.6621	45.98053
136	黑龙江	大兴安岭地区漠河县北极镇洛古河村	乡镇	122.8693	53.36525
137	黑龙江	佳木斯市桦川县星火朝鲜族乡星火村	村庄	130.5636	46.88086
138	黑龙江	七台河市勃利县勃利镇元明村	村庄	130.5019	45.79667
139	黑龙江	牡丹江市海林市横道河子镇七里地村	村庄	129.0925	44.81597
140	黑龙江	齐齐哈尔市讷河市兴旺鄂温克族乡索伦	村庄	124.5072	48.12073

141	黑龙江	伊春市铁力市年丰朝鲜族乡长山村	村庄	128.0247	46.93328
142	黑龙江	黑河市五大连池市朝阳乡边河村	村庄	126.3852	48.92668
143	黑龙江	鸡西市密山市白鱼湾镇湖沿村	村庄	132.1391	45.33215
144	黑龙江	黑河市爱辉区新生乡新生村	村庄	126.7911	50.50896
145	黑龙江	鹤岗市萝北县东明乡红光村	村庄	130.8119	47.56107
146	黑龙江	齐齐哈尔市甘南县兴十四镇兴十四村	村庄	123.6938	47.86856
147	黑龙江	大庆市杜蒙县胡吉吐莫镇东吐莫村	村庄	124.0672	46.53168
148	黑龙江	绥化市兰西县兰西镇永久村	村庄	126.3177	46.207
149	黑龙江	佳木斯市抚远市乌苏镇抓吉赫哲族村	村庄	134.653	48.17177
150	黑龙江	伊春市大箐山县朗乡镇达里村	村庄	128.7619	46.95963
151	黑龙江	佳木斯市汤原县汤旺朝鲜族乡金星村	村庄	129.7441	46.60587
152	黑龙江	兴安岭地区呼玛县白银纳鄂伦春族乡白银纟	村庄	125.9159	52.41591
153	上海	金山区廊下镇山塘村	村庄	121.1597	30.7782
154	上海	崇明区绿华镇绿港村	村庄	121.2171	31.75107
155	上海	浦东新区大团镇赵桥村	村庄	121.7346	30.99057
156	上海	青浦区朱家角镇张马村	村庄	121.0804	31.0448
157	上海	宝山区罗泾镇塘湾村	村庄	121.3116	31.48571
158	上海	崇明区横沙乡丰乐村	村庄	121.8461	31.32712
159	上海	崇明区陈家镇瀛东村	村庄	121.839	31.46576
160	上海	金山区朱泾镇待泾村	村庄	121.1197	30.88451
161	上海	宝山区罗泾镇海星村	村庄	121.3442	31.48583
162	上海	金山区枫泾镇中洪村	村庄	121.001	30.92657
163	上海	浦东新区祝桥镇邓三村	村庄	121.7561	31.13693
164	江苏	南京市江宁区横溪街道石塘村	村庄	118.6915	31.71973
165	江苏	常州市溧阳市溧城镇礼诗圩村	村庄	119.5383	31.42817
166	江苏	泰州市姜堰区三水街道小杨村	村庄	120.0728	32.57385
167	江苏	镇江市句容市茅山镇丁庄村	村庄	119.2506	31.89354
168	江苏	苏州市高新区通安镇树山村	村庄	120.4527	31.36364
169	江苏	南通市如东县栟茶镇三园村	村庄	120.8907	32.51824
170	江苏	盐城市东台市五烈镇甘港村	村庄	120.267	32.91621
171	江苏	徐州市铜山区汉王镇汉王村	村庄	117.0824	34.20006
172	江苏	泰州市兴化市千垛镇东罗村	村庄	119.7947	33.04413
173	江苏	常州市武进区雪堰镇城西回民村	村庄	120.0362	31.51438
174	江苏	连云港市连云区西连岛村	购物	119.444	34.77349
175	江苏	南京市溧水区白马镇李巷村	村庄	119.1374	31.52503
176	江苏	镇江市丹徒区江心洲生态农业园区五套村	NoClass	119.5889	32.21512
177	江苏	徐州市铜山区柳泉镇北村	村庄	117.2708	34.50279
178	江苏	南京市高淳区东坝街道三条垄田园慢村	村庄	119.0574	31.37304
179	江苏	无锡市滨湖区马山街道群丰社区	医疗	120.1016	31.42572
180	江苏	无锡市江阴市华士镇华西新市村	村庄	120.4318	31.83295
181	江苏	常州市溧阳市南渡镇庆丰村	村庄	119.2765	31.44733
182	江苏	南通市如皋市城北街道平园池村	村庄	120.5022	32.4501
183	江苏	连云港市灌云县伊山镇川星村	村庄	119.2946	34.30706
184	江苏	徐州市睢宁县姚集镇高党村	村庄	117.8437	34.0879
185	江苏	无锡市宜兴市西渚镇白塔村	村庄	119.5426	31.29657
186	江苏	盐城市盐都区郭猛镇杨侍村	村庄	120.0323	33.28141
187	江苏	淮安市金湖县前锋镇白马湖村	村庄	119.16	33.18654

188	江苏	南京市江宁区谷里街道双塘社区大塘金村	NoClass	118.7103	31.85799
189	江苏	徐州市贾汪区茱萸山街道磨石塘村	区县	117.4571	34.43649
190	浙江	湖州市德清县莫干山镇劳岭村	村庄	119.894	30.58406
191	浙江	湖州市安吉县递铺街道鲁家村	村庄	119.7448	30.68242
192	浙江	金华市磐安县尖山镇乌石村	村庄	120.732	29.23959
193	浙江	衢州市江山市石门镇清漾村	旅游景点	118.5793	28.57353
194	浙江	衢州市江山市廿八都镇浔里村	村庄	118.477	28.29577
195	浙江	丽水市缙云县新建镇河阳村	村庄	120.0052	28.72756
196	浙江	杭州市西湖区转塘街道上城埭村	村庄	120.0393	30.18214
197	浙江	宁波市象山县墙头镇方家岙村	村庄	121.7805	29.45297
198	浙江	湖州市安吉县灵峰街道横山坞村	村庄	119.6129	30.61599
199	浙江	杭州市建德市大慈岩镇新叶村	旅游景点	119.3304	29.33228
200	浙江	温州市文成县南田镇武阳村	村庄	119.947	27.95799
201	浙江	湖州市南浔区和孚镇荻港村	村庄	120.1554	30.76266
202	浙江	台州市天台县赤城街道塔后村	村庄	121.0122	29.17278
203	浙江	舟山市定海区干览镇新建村	村庄	122.1346	30.0914
204	浙江	台州市三门县横渡镇岩下潘村	村庄	121.4493	28.98377
205	浙江	绍兴市新昌县镜岭镇外婆坑村	村庄	120.7149	29.32876
206	浙江	金华市浦江县虞宅乡新光村	村庄	119.8163	29.56869
207	浙江	宁波市宁海县桥头胡街道双林村	村庄	121.5435	29.37532
208	浙江	丽水市松阳县大东坝镇茶排村	乡镇	119.4815	28.34616
209	浙江	杭州市临安区高虹镇石门村	村庄	119.6647	30.36802
210	浙江	金华市东阳市南马镇花园村	村庄	120.2186	29.12019
211	浙江	绍兴市上虞区岭南乡东澄村	村庄	120.9648	29.75436
212	浙江	绍兴市柯桥区漓渚镇棠棣村	村庄	120.4367	29.95652
213	浙江	温州市永嘉县岩头镇苍坡村	村庄	120.746	28.3635
214	浙江	宁波市宁海县大佳何镇葛家村	村庄	121.602	29.42772
215	浙江	嘉兴市海宁市丁桥镇新仓村	村庄	120.694	30.40411
216	安徽	黄山市徽州区呈坎镇呈坎村	旅游景点	118.2793	29.92439
217	安徽	六安市金寨县花石乡大湾村	村庄	115.6806	31.33856
218	安徽	黄山市黟县西递镇西递村	村庄	117.9907	29.90585
219	安徽	合肥市长丰县杨庙镇马郢社区	村庄	117.0053	32.20605
220	安徽	芜湖市南陵县烟墩镇霭里村	村庄	118.1432	30.69043
221	安徽	六安市霍山县磨子潭镇堆谷山村	村庄	116.2873	31.13521
222	安徽	铜陵市义安区西联镇犁桥村	道路	117.8986	31.01934
223	安徽	亳州市谯城区古井镇药王村	乡镇	115.6644	33.99105
224	安徽	安庆市潜山市官庄镇官庄村	村庄	116.6063	31.02283
225	安徽	黄山市休宁县溪口镇祖源村	村庄	118.0306	29.66177
226	安徽	马鞍山市当涂县护河镇桃花村	乡镇	118.5986	31.50585
227	安徽	六安市霍山县太阳乡金竹坪村	村庄	116.1511	31.13178
228	安徽	滁州市明光市张八岭镇柴郢村	村庄	118.3032	32.58757
229	安徽	黄山市黄山区汤口镇山岔村	村庄	118.2188	30.10309
230	安徽	黄山市黟县宏村镇塔川村	乡镇	117.985	30.00523
231	安徽	宣城市绩溪县家朋乡尚村	村庄	118.7916	30.21189
232	安徽	六安市金安区张店镇洪山村	村庄	116.5566	31.49304
233	安徽	宣城市旌德县白地镇江村	旅游景点	118.3898	30.21784
234	安徽	合肥市庐江县万山镇长冲村	乡镇	117.176	31.32936

235	安徽	芜湖市芜湖县红杨镇珩琅山村	乡镇	118.5525	31.06713
236	安徽	淮北市烈山区烈山镇榴园村	村庄	116.9476	33.92941
237	安徽	亳州市涡阳县曹市镇辉山村	村庄	116.4962	33.49274
238	福建	泉州市晋江市金井镇围头村	村庄	118.5765	24.52897
239	福建	漳州市华安县新圩镇官畲村	村庄	117.5777	24.92645
240	福建	厦门市海沧区海沧街道青礁村	村庄	117.9607	24.47498
241	福建	莆田市涵江区白沙镇坪盘村	村庄	118.968	25.54954
242	福建	平潭综合实验区苏平片区上攀村	NoClass	119.7059	25.48473
243	福建	莆田市湄洲岛湄洲镇下山村	村庄	119.1142	25.04011
244	福建	宁德市古田县城东街道桃溪村	村庄	118.7066	26.65542
245	福建	漳州市平和县芦溪镇蕉路村	村庄	117.0484	24.47716
246	福建	南平市邵武市和平镇和平村	村庄	117.2976	27.16222
247	福建	龙岩市武平县城厢镇云寨村	乡镇	116.1001	25.09991
248	福建	福州市罗源县霍口畲族乡福湖村	村庄	119.2849	26.44009
249	福建	龙岩市新罗区小池镇培斜村	村庄	116.8497	25.11679
250	福建	漳州市南靖县书洋镇塔下村	村庄	117.0456	24.61971
251	福建	泉州市德化县国宝乡佛岭村	村庄	118.1787	25.60536
252	福建	三明市清流县林畲镇林畲村	村庄	117.0717	26.31901
253	福建	三明市大田县济阳乡济中村	村庄	117.9526	25.53684
254	福建	宁德市屏南县熙岭乡龙潭村	村庄	119.0695	26.77521
255	福建	三明市泰宁县上青乡崇际村	乡镇	117.1687	27.03956
256	福建	南平市武夷山市五夫镇兴贤村	村庄	118.2057	27.61543
257	福建	龙岩市永定区陈东乡岩太村	村庄	116.9665	24.75664
258	福建	厦门市同安区莲花镇军营村	村庄	117.9733	24.84874
259	福建	南平市建瓯市小松镇湖头村	村庄	118.3588	27.12245
260	福建	福州市永泰县嵩口镇月洲村	村庄	118.6251	25.81744
261	福建	福州市永泰县梧桐镇春光村	村庄	118.7642	25.73132
262	福建	龙岩市武平县万安镇捷文村	村庄	116.0489	25.17218
263	福建	南平市武夷山市兴田镇南源岭村	村庄	117.9849	27.6131
264	江西	景德镇市浮梁县瑶里镇瑶里村	村庄	117.5783	29.54789
265	江西	南昌市安义县石鼻镇罗田村	村庄	115.6013	28.75159
266	江西	萍乡市芦溪县宣风镇竹垣村	村庄	114.1717	27.72515
267	江西	萍乡市湘东区麻山镇幸福村	村庄	113.8006	27.56061
268	江西	吉安市万安县高陂镇高陂村	村庄	114.632	26.6279
269	江西	抚州市南丰县市山镇包坊村	村庄	116.4891	27.23626
270	江西	九江市武宁县罗坪镇长水村	村庄	115.2615	29.17266
271	江西	明月山温泉风景名胜区（袁州区）温汤镇	旅游景点	114.2873	27.60298
272	江西	吉安市安福县章庄乡章庄村	村庄	114.3601	27.52585
273	江西	吉安市永新县高市乡滨江村（洲塘书画村）	旅游景点	114.1869	27.02567
274	江西	抚州市广昌县驿前镇姚西村	村庄	116.2924	26.54247
275	江西	上饶市德兴市香屯街道杨家湾村楼上楼村	村庄	117.5409	28.99944
276	江西	上饶市婺源县紫阳镇考水村	村庄	117.7441	29.27278
277	江西	上饶市婺源县溪头乡西岸村江岭村	旅游景点	118.0285	29.45567
278	江西	赣州市上犹县梅水乡园村村	生活服务	114.4317	25.8758
279	江西	明月山温泉风景名胜区（袁州区）洪江镇	旅游景点	114.2873	27.60298
280	江西	赣州市大余县新城镇周屋村	村庄	114.6127	25.52432
281	江西	抚州市资溪县乌石镇草坪村	村庄	116.9845	27.62866

282	江西	抚州市资溪县马头山镇永胜村	村庄	117.1141	27.82418
283	江西	鹰潭市贵溪市雷溪镇南山村	村庄	117.2017	28.2267
284	江西	赣州市石城县琴江镇大畲村	村庄	116.3779	26.2988
285	江西	上饶市婺源县蚺城街道上梅洲村塘村	村庄	117.8648	29.22129
286	江西	九江市修水县杭口镇双井村	村庄	114.4846	29.04915
287	江西	吉安市井冈山市茅坪镇神山村	村庄	114.0986	26.64726
288	江西	吉安市井冈山市黄坳乡黄坳村	乡镇	114.2344	26.4816
289	山东	济南市长清区万德街道马套村	村庄	117.0071	36.27178
290	山东	临沂市沂南县马牧池乡常山庄村	村庄	118.3029	35.67171
291	山东	临沂市兰陵县卞庄街道代村	村庄	118.0332	34.84012
292	山东	临沂市蒙阴县岱崮镇笊篱坪村	村庄	118.1366	35.9332
293	山东	威海市荣成市俚岛镇烟墩角村	村庄	122.5578	37.29386
294	山东	临沂市平邑县地方镇九间棚村	村庄	117.7358	35.29575
295	山东	威海市环翠区张村镇王家疃村	村庄	122.0754	37.47171
296	山东	潍坊市寒亭区杨家埠旅游开发区西杨家埠	旅游景点	119.216	36.75679
297	山东	泰安市肥城市孙伯镇五埠村	村庄	116.6445	35.99391
298	山东	照市山海天旅游度假区卧龙山街道李家台	旅游景点	119.5751	35.44799
299	山东	临沂市沂水县院东头镇四门洞村	旅游景点	118.4465	35.71489
300	山东	潍坊市临朐县五井镇隐士村	村庄	118.3225	36.44487
301	山东	济宁市泗水县圣水峪镇东仲都村	村庄	117.2707	35.58662
302	山东	滨州市滨城区里则街道西纸坊村	村庄	117.8834	37.27177
303	山东	淄博市淄川区昆仑镇牛记庵村	乡镇	117.9129	36.58868
304	山东	潍坊市坊子区坊安街道洼里村	村庄	119.2047	36.56643
305	山东	威海市文登区高村镇慈口观村	村庄	122.1537	37.11371
306	山东	济南市南部山区西营街道黄鹿泉村	村庄	117.2251	36.53913
307	山东	菏泽市巨野县核桃园镇前王庄村	村庄	116.2578	35.25495
308	山东	泰安市岱岳区道朗镇东西门村	村庄	116.9474	36.25358
309	山东	青岛市崂山区沙子口街道东麦窑社区	地产小区	120.6103	36.12725
310	山东	潍坊市青州市王坟镇胡林古村	村庄	118.2873	36.49515
311	山东	枣庄市山亭区徐庄镇葫芦套村	村庄	117.5916	35.0381
312	山东	济宁市曲阜市石门山镇石门山庄村	乡镇	117.0592	35.71325
313	河南	郑州市新密市米村镇朱家庵村	村庄	113.2394	34.55825
314	河南	信阳市罗山县铁铺镇何家冲村	村庄	114.311	31.81437
315	河南	商丘市民权县北关镇王公庄村	村庄	115.2882	34.82672
316	河南	信阳市新县田铺乡田铺大塆村	乡镇	114.978	31.5392
317	河南	巩义市竹林镇石鼓村	乡镇	113.1191	34.70113
318	河南	驻马店市遂平县嵖岈山镇红石崖村	乡镇	113.7458	33.20271
319	河南	郑州市二七区侯寨乡樱桃沟社区	旅游景点	113.583	34.61911
320	河南	漯河市临颍县城关镇南街村	旅游景点	113.9516	33.80178
321	河南	鹤壁市淇县灵山街道凉水泉村	村庄	114.0952	35.69014
322	河南	安阳市林州市石板岩镇高家台村	村庄	113.6835	36.11342
323	河南	许昌市襄城县紫云镇雷洞村	村庄	113.4049	33.78857
324	河南	安阳市林州市黄华镇庙荒村	村庄	113.7416	36.07283
325	河南	南阳市南召县云阳镇铁佛寺村	村庄	112.711	33.40797
326	河南	洛阳市栾川县庙子镇庄子村	村庄	111.7351	33.73142
327	河南	焦作市孟州市西虢镇莫沟村	村庄	112.6484	34.91757
328	河南	南阳市淅川县仓房镇磨沟村	村庄	111.4423	32.77926

329	河南	焦作市修武县云台山镇岸上村	村庄	113.3737	35.4193
330	河南	三门峡市渑池县段村乡赵沟村	村庄	111.8904	34.94552
331	河南	洛阳市嵩县黄庄乡三合村	村庄	112.1916	34.01425
332	河南	洛阳市栾川县陶湾镇协心村	村庄	111.4486	33.81714
333	河南	信阳市新县周河乡西河村	村庄	115.0219	31.63162
334	湖北	宜昌市夷陵区太平溪镇许家冲村	村庄	111.0221	30.84289
335	湖北	武汉市黄陂区姚家集街道杜堂村	村庄	114.382	31.23759
336	湖北	鄂州市梁子湖区涂家垴镇万秀村	村庄	114.5993	30.13248
337	湖北	武汉市蔡甸区大集镇天星村	村庄	114.0484	30.50379
338	湖北	十堰市郧西县上津镇津城村	村庄	110.0393	33.13835
339	湖北	武汉市黄陂区木兰乡双泉村	乡镇	114.4571	31.10195
340	湖北	襄阳市谷城县五山镇堰河村	村庄	111.3234	32.30055
341	湖北	宜昌市长阳县龙舟坪镇郑家榜村	村庄	110.9981	30.53338
342	湖北	宜昌市秭归县屈原镇西陵峡村	村庄	110.8249	30.90661
343	湖北	襄阳市老河口市仙人渡镇李家染坊村	村庄	111.7719	32.28135
344	湖北	宜昌市宜都市高坝洲镇青林寺村	村庄	111.3551	30.48359
345	湖北	武汉市江夏区五里界街道童周岭村	村庄	114.4356	30.28593
346	湖北	鄂州市华容区段店镇武圣村	村庄	114.7669	30.55047
347	湖北	宜昌市夷陵区龙泉镇青龙村	村庄	111.5228	30.74745
348	湖北	十堰市郧阳区柳陂镇龙韵村	村庄	110.7423	32.8429
349	湖北	荆门市钟祥市客店镇马湾村	村庄	112.8642	31.36464
350	湖北	十堰市郧阳区茶店镇樱桃沟村	村庄	110.7654	32.76502
351	湖北	荆州市洪湖市老湾回族乡珂里村	村庄	113.655	29.99748
352	湖北	荆州市石首市团山寺镇过脉岭村	村庄	112.3053	29.58025
353	湖北	孝感市安陆市烟店镇碧山村	村庄	113.5756	31.32803
354	湖北	襄阳市保康县店垭镇格栏坪村	村庄	111.3748	31.3966
355	湖北	黄石市阳新县兴国镇南市村	村庄	115.19	29.79035
356	湖北	宜昌市五峰县长乐坪镇白岩坪村	村庄	110.9146	30.16992
357	湖北	黄石市大冶市保安镇沼山村	村庄	114.7199	30.14707
358	湖北	十堰市郧西县涧池乡下营村	村庄	110.304	32.88958
359	湖北	土家族苗族自治州恩施市盛家坝镇二官	村庄	109.234	30.05885
360	湖北	宜昌市宜都市枝城镇全心畈村	乡镇	111.4951	30.3004
361	湖南	永州市宁远县湾井镇下灌村	村庄	112.0084	25.47005
362	湖南	怀化市通道侗族自治县坪坦乡皇都村	旅游景点	109.7218	26.10862
363	湖南	常德市桃源县枫树维回乡维回新村	村庄	111.497	29.02768
364	湖南	株洲市攸县酒埠江镇酒仙湖村	村庄	113.5635	27.21973
365	湖南	永州市双牌县茶林镇桐子坳村	村庄	111.8468	26.04882
366	湖南	益阳市桃江县大栗港镇刘家村	村庄	111.8606	28.47256
367	湖南	衡阳市南岳区南岳镇红星村	村庄	112.7516	27.21673
368	湖南	株洲市炎陵县十都镇密花村	村庄	113.9863	26.50406
369	湖南	岳阳市屈原区河市镇三和村	村庄	113.0302	28.86776
370	湖南	常德市津市市毛里湖镇青苗社区	村庄	111.9983	29.3916
371	湖南	西土家族苗族自治州永顺县灵溪镇司城	村庄	109.9713	28.99621
372	湖南	长沙市长沙县开慧镇锡福村	村庄	113.2984	28.58947
373	湖南	益阳市资阳区长春镇紫薇村	村庄	112.3096	28.66539
374	湖南	邵阳市新宁县崀山镇石田村	村庄	110.7596	26.3521
375	湖南	湘潭市韶山市韶山乡韶山村	村庄	112.4843	27.90673

376	湖南	岳阳市临湘市羊楼司镇龙窖山村	区县	113.444	29.47962
377	湖南	郴州市安仁县永乐江镇山塘村	村庄	113.323	26.73975
378	湖南	永州市祁阳县茅竹镇三家村	村庄	111.7805	26.56607
379	湖南	长沙市浏阳市张坊镇田溪村	村庄	114.1574	28.36298
380	湖南	怀化市鹤城区黄岩区大坪村	区县	110.0352	27.5821
381	湖南	张家界市永定区尹家溪镇马儿山村	乡镇	110.4136	29.13763
382	湖南	张家界市武陵源区协合乡龙尾巴村	村庄	110.4707	29.31904
383	湖南	张家界市武陵源区天子山街道泗南峪社区	村庄	110.4471	29.41005
384	广东	珠海市斗门区斗门镇南门村	村庄	113.1845	22.23829
385	广东	梅州市梅县区雁洋镇长教村	村庄	116.3692	24.37781
386	广东	梅州市平远县泗水镇梅畲村	村庄	116.0357	24.72133
387	广东	东莞市寮步镇陈家埔村	村庄	113.8533	22.99897
388	广东	清远市英德市九龙镇河头村	村庄	112.9277	24.12166
389	广东	惠州市博罗县横河镇上良村	村庄	114.1985	23.30931
390	广东	揭阳市揭西县金和镇山湖村	村庄	116.0802	23.44026
391	广东	湛江市雷州市龙门镇足荣村	村庄	109.9308	20.62696
392	广东	广州市从化区温泉镇南平村	村庄	113.7119	23.55083
393	广东	肇庆市德庆县官圩镇金林村	村庄	111.8082	23.28938
394	广东	江门市台山市海宴镇五丰村	村庄	112.5431	21.78398
395	广东	广州市从化区吕田镇莲麻村	村庄	114.0044	23.92098
396	广东	阳江市阳东区东平镇大澳渔村	旅游景点	112.2439	21.71123
397	广东	汕头市澄海区隆都镇前美村	村庄	116.7408	23.56903
398	广东	韶关市南雄市珠玑镇灵潭村	村庄	114.3859	25.25539
399	广东	佛山市南海区西樵镇上金瓯村松塘村	村庄	112.9077	22.9898
400	广东	肇庆市四会市江谷镇老泗塘村	村庄	112.6823	23.52678
401	广东	广州市番禺区石楼镇大岭村	村庄	113.472	22.98781
402	广东	江门市开平市塘口镇强亚村	村庄	112.5763	22.37225
403	广东	茂名市高州市根子镇柏桥村	村庄	111.0121	21.77151
404	广东	河源市东源县康禾镇仙坑村	村庄	115.0886	23.84828
405	广东	江门市台山市水步镇草坪村	乡镇	112.7943	22.33042
406	广西	柳州市融水苗族自治县融水镇新国村	村庄	109.2417	25.10439
407	广西	南宁市西乡塘区石埠街道忠良村	村庄	108.1559	22.77173
408	广西	崇左市宁明县城中镇耀达村	村庄	107.0055	22.25453
409	广西	百色市田东县祥周镇模范村	村庄	106.9739	23.61075
410	广西	桂林市恭城瑶族自治县莲花镇红岩村	村庄	110.8562	24.73277
411	广西	崇左市江州区新和镇卜花村	村庄	107.2024	22.57985
412	广西	桂林市灌阳县新街镇江口村	村庄	111.1102	25.41694
413	广西	柳州市三江侗族自治县丹洲镇丹洲村	村庄	109.4401	25.37412
414	广西	南宁市马山县古零镇羊山村三甲屯	村庄	108.2691	23.65635
415	广西	来宾市金秀瑶族自治县长垌乡平道村	村庄	110.0858	24.09578
416	广西	梧州市藤县象棋镇道家村	村庄	110.689	23.12953
417	广西	桂林市阳朔县阳朔镇骥马村	村庄	110.4534	24.7696
418	广西	柳州市鹿寨县中渡镇大兆村	村庄	109.6855	24.71044
419	广西	梧州市蒙山县新圩镇古定村	村庄	110.3968	24.30388
420	广西	桂林市阳朔县阳朔镇鸡窝渡村	村庄	110.4902	24.72778
421	广西	来宾市金秀瑶族自治县长垌乡滴水村	村庄	110.1188	24.02483
422	广西	百色市靖西市新靖镇旧州村	村庄	106.4137	23.06095

423	广西	北海市海城区地角街道新营社区流下村	村庄	109.0585	21.45784
424	广西	百色市德保县城关镇那温村	村庄	106.684	23.31664
425	广西	玉林市陆川县沙坡镇高庆村	村庄	110.3648	22.30597
426	广西	防城港市东兴市江平镇交东村	村庄	108.2044	21.61132
427	广西	贺州市平桂区沙田镇龙井村	村庄	111.4622	24.33884
428	海南	三亚市吉阳区博后村	村庄	109.6046	18.24426
429	海南	三亚市吉阳区大茅村	村庄	109.6355	18.34681
430	海南	琼海市嘉积镇官塘村北仍村	村庄	110.4124	19.21155
431	海南	三亚市海棠区湾坡村	村庄	109.6919	18.31691
432	海南	琼海市博鳌镇沙美村	村庄	110.552	19.1059
433	海南	儋州市那大镇石屋村	村庄	109.5445	19.55537
434	海南	琼海市博鳌镇朝烈村南强村	村庄	110.5627	19.15944
435	海南	海口市秀英区石山镇施茶村	村庄	110.2297	19.93017
436	海南	文昌市龙楼镇好圣村	村庄	111.0028	19.65216
437	海南	儋州市那大镇屋基村	村庄	109.5328	19.63861
438	海南	澄迈县大丰镇大丰村	村庄	110.0416	19.9135
439	海南	陵水黎族自治县本号镇小妹村	乡镇	109.9584	18.61075
440	海南	文昌市东路镇葫芦村	村庄	110.6778	19.789
441	海南	三亚市天涯区文门村	乡镇	109.3231	18.3091
442	海南	海口市龙华区新坡镇仁里村	村庄	110.3289	19.79752
443	海南	海口市琼山区红旗镇苏寻三村泮边村	村庄	110.5407	19.86404
444	重庆	武隆区后坪苗族土家族乡文凤村	村庄	108.0308	29.60145
445	重庆	武隆区芙蓉街道堰塘村	村庄	107.7844	29.34229
446	重庆	石柱土家族自治县中益乡华溪村	旅游景点	108.3432	30.10235
447	重庆	铜梁区土桥镇六赢村	村庄	106.0191	29.79713
448	重庆	巴南区二圣镇集体村	村庄	106.8056	29.46398
449	重庆	巫溪县红池坝镇茶山村	村庄	108.9472	31.53041
450	重庆	梁平区竹山镇猎神村	村庄	107.6079	30.75909
451	重庆	丰都县双路镇莲花洞村	村庄	107.8382	29.84985
452	重庆	綦江区永城镇中华村	村庄	106.8198	29.02047
453	重庆	涪陵区大木乡迎新社区	村庄	107.6642	29.62349
454	重庆	酉阳土家族苗族自治县板溪镇扎营村	村庄	108.7886	28.71564
455	重庆	黔江区小南海镇新建村	村庄	108.6843	29.65148
456	重庆	南川区木凉镇汉场坝村	村庄	106.9935	29.21201
457	重庆	南岸区南山街道放牛村	村庄	106.646	29.57449
458	重庆	荣昌区仁义镇瑶山社区	村庄	105.5131	29.50207
459	重庆	彭水苗族土家族自治县润溪乡樱桃井村	村庄	107.9715	29.17687
460	重庆	巫山县两坪乡朝元村	村庄	110.005	31.13971
461	重庆	长寿区龙河镇保合村	村庄	107.1946	29.98755
462	重庆	北碚区东阳街道西山坪村	村庄	106.4261	29.88586
463	重庆	巫山县曲尺乡柑园村	乡镇	109.7456	31.03539
464	四川	成都市崇州市白头镇五星村	村庄	103.6214	30.60377
465	四川	[illegible]坝藏族羌族自治州黑水县沙石多乡羊茸[illegible]	村庄	102.8557	32.11205
466	四川	泸州市纳溪区大渡口镇凤凰湖村	旅游景点	105.3104	28.7031
467	四川	广元市利州区白朝乡月坝村	村庄	105.4184	32.38059
468	四川	成都市龙泉驿区山泉镇桃源村	村庄	104.3194	30.55259
469	四川	阿坝藏族羌族自治州理县桃坪镇桃坪村	村庄	103.4597	31.55692

470	四川	成都市彭州市桂花镇蟠龙村	村庄	103.7828	31.11073
471	四川	攀枝花市米易县新山傈僳族乡新山村	村庄	102.1659	26.84124
472	四川	凉山彝族自治州德昌县德州镇角半村	村庄	102.1217	27.42421
473	四川	孜藏族自治州丹巴县墨尔多山镇基卡依	村庄	101.9251	30.89074
474	四川	资阳市乐至县劳动镇旧居村	村庄	104.9582	30.38051
475	四川	广安市武胜县飞龙镇高洞村	村庄	106.373	30.38589
476	四川	广元市青川县青溪镇阴平村	村庄	104.8251	32.4734
477	四川	宜宾市筠连县腾达镇春风村	村庄	104.5586	28.166
478	四川	广安市广安区协兴镇牌坊社区	乡镇	106.6368	30.53719
479	四川	成都市都江堰市龙池镇飞虹社区	乡镇	103.5831	31.02342
480	四川	绵阳市涪城区杨家镇杨家社区	乡镇	104.717	31.34227
481	四川	南充市蓬安县相如街道油房沟社区	村庄	106.3537	31.01352
482	四川	遂宁市大英县卓筒井镇为干屏村	村庄	105.1688	30.49161
483	四川	乐山市峨眉山市胜利街道月南村	村庄	103.5353	29.59108
484	四川	德阳市绵竹市九龙镇新龙村	村庄	104.1475	31.39541
485	四川	广元市青川县乔庄镇张家村	村庄	105.2018	32.55734
486	四川	成都市都江堰市青城山镇泰安社区	地产小区	103.4871	30.91647
487	贵州	六盘水市盘州市普古彝族苗族乡舍烹村	村庄	104.8712	26.07865
488	贵州	东南苗族侗族自治州黎平县肇兴镇肇兴	村庄	109.1769	25.91033
489	贵州	贵阳市乌当区偏坡布依族乡偏坡村	村庄	106.9237	26.65613
490	贵州	东南苗族侗族自治州榕江县平阳乡丹江	村庄	108.3405	26.3411
491	贵州	铜仁市玉屏侗族自治县田坪镇田坪村	村庄	109.113	27.44228
492	贵州	盘水市水城县营盘苗族彝族白族乡高峰	村庄	105.3996	26.23029
493	贵州	南布依族苗族自治州贵定县盘江镇音寨	村庄	107.1693	26.47211
494	贵州	盘水市六枝特区落别布依族彝族乡牛角	乡镇	105.5926	26.11821
495	贵州	遵义市赤水市复兴镇凯旋村	村庄	105.7703	28.45799
496	贵州	西南布依族苗族自治州贞丰县者相镇纳孔	村庄	105.6323	25.51834
497	贵州	遵义市凤冈县永安镇田坝村	村庄	107.57	28.08188
498	贵州	铜仁市石阡县坪山仡佬族侗族乡佛顶山村	乡镇	108.1293	27.41733
499	贵州	六盘水市盘州市两河街道岩脚村	村庄	104.6225	25.87136
500	贵州	南布依族苗族自治州都匀市毛尖镇坪阳	村庄	107.3604	26.255
501	贵州	铜仁市松桃苗族自治县正大镇薅菜村	村庄	109.2251	27.95289
502	贵州	西南布依族苗族自治州兴仁市屯脚镇鲤鱼	村庄	105.3128	25.39492
503	贵州	遵义市湄潭县兴隆镇龙凤村	村庄	107.5418	27.72899
504	贵州	毕节市织金县官寨苗族乡屯上村	村庄	105.8934	26.78865
505	贵州	贵阳市花溪区青岩镇龙井村	村庄	106.6701	26.3366
506	贵州	东南苗族侗族自治州从江县丙妹镇岜沙	NoClass	108.8623	25.72324
507	贵州	顺市镇宁布依族苗族自治县宁西街道高荡	村庄	105.69	26.07577
508	贵州	市金海湖新区响水白族彝族仡佬族乡青	村庄	105.5423	27.28671
509	贵州	布依族苗族自治州兴义市万峰林街道下	村庄	104.9237	24.9728
510	贵州	贵阳市花溪区高坡苗族乡扰绕村	村庄	106.7914	26.31135
511	贵州	遵义市赤水市天台镇凤凰村	村庄	105.8163	28.55394
512	贵州	安顺市西秀区双堡镇大坝村	村庄	106.1077	26.15446
513	云南	丽江市玉龙纳西族自治县拉市镇美泉村	乡镇	100.1467	26.85191
514	云南	河哈尼族彝族自治州弥勒市西三镇可邑	旅游景点	103.3647	24.51661
515	云南	临沧市沧源佤族自治县勐角乡翁丁村	村庄	99.16327	23.28105
516	云南	保山市腾冲市清水乡中寨司莫拉佤族村	村庄	98.46729	24.92959

517	云南	昆明市石林彝族自治县圭山镇大糯黑村	村庄	103.5265	24.66604
518	云南	丽江市玉龙纳西族自治县白沙镇玉湖村	乡镇	100.2171	26.95744
519	云南	保山市腾冲市固东镇江东社区	地产小区	98.52118	25.30993
520	云南	版纳傣族自治州景洪市基诺乡巴亚村巴	村庄	101.0044	22.09634
521	云南	昭通市彝良县小草坝镇小草坝村	村庄	104.2582	27.77242
522	云南	玉溪市澄江市右所镇小湾村	村庄	102.9534	24.58053
523	云南	曲靖市师宗县五龙乡狗街村	村庄	104.2849	24.62287
524	云南	理白族自治州洱源县凤羽镇江登村佛堂	村庄	99.96135	25.99209
525	云南	楚雄彝族自治州南华县龙川镇岔河村	村庄	101.2669	25.32046
526	云南	曲靖市会泽县娜姑镇白雾村	村庄	103.1917	26.48679
527	云南	临沧市凤庆县凤山镇安石村	村庄	99.8743	24.63439
528	云南	德宏傣族景颇族自治州芒市芒市镇回贤村	村庄	98.63811	24.3964
529	云南	河哈尼族彝族自治州元阳县新街镇阿者科	村庄	102.8185	23.09095
530	云南	江傈僳族自治州贡山县丙中洛镇秋那桶	村庄	98.57365	28.09627
531	云南	昆明市宜良县九乡彝族回族乡麦地冲村	村庄	103.3933	25.12187
532	云南	澜沧拉祜族自治县酒井哈尼族乡勐根村老	村庄	99.97114	22.33427
533	云南	洱市西盟佤族自治县勐卡镇马散村永俄	NoClass	99.43182	22.7714
534	云南	迪庆藏族自治州香格里拉市尼西乡汤堆村	乡镇	99.50543	28.05095
535	云南	藏族自治州维西傈僳族自治县塔城镇启	村庄	99.43856	27.57771
536	西藏	拉萨市达孜区邦堆乡叶巴村	村庄	91.28262	29.69451
537	西藏	拉萨市堆龙德庆区乃琼镇波玛村	村庄	90.87955	29.68919
538	西藏	林芝市巴宜区鲁朗镇东巴才村	村庄	94.73631	29.673
539	西藏	拉萨市尼木县吞巴乡吞达村	乡镇	90.28533	29.33896
540	西藏	林芝市工布江达县错高乡错高村	村庄	94.0323	30.06578
541	西藏	日喀则市亚东县康布乡上康布村	村庄	88.98816	27.77876
542	西藏	拉萨市城关区柳梧新区达东村	地产小区	91.09574	29.60083
543	西藏	那曲市班戈县青龙乡东嘎村	村庄	90.79451	31.0882
544	西藏	拉萨市城关区夺底乡维巴村	村庄	91.15494	29.72976
545	西藏	林芝市巴宜区林芝镇立定村	村庄	94.47643	29.4525
546	西藏	拉萨市曲水县曲水镇俊巴村	乡镇	90.74097	29.3527
547	西藏	昌都市江达县同普乡夏乌村	村庄	98.37731	31.5906
548	西藏	拉萨市城关区娘热乡加尔西村	政府机构	91.10223	29.71369
549	西藏	阿里地区普兰县普兰镇科迦村	村庄	81.27453	30.19766
550	西藏	山南市桑日县增期乡雪巴村	村庄	92.316	29.37738
551	西藏	日喀则市亚东县下亚东乡夏日村	乡镇	88.9281	27.42965
552	西藏	昌都市芒康县嘎托镇觉龙村	乡镇	98.59448	29.68199
553	西藏	阿里地区札达县托林镇扎布让村	乡镇	79.61549	31.37481
554	西藏	山南市错那县勒布乡勒村	区县	91.95623	27.99479
555	西藏	日喀则市仁布县切瓦乡嘎布久嘎村	区县	89.84029	29.23412
556	西藏	山南市乃东区昌珠镇扎西曲登社区	乡镇	91.77151	29.1902
557	陕西	西安市长安区王曲街道南堡寨村	村庄	108.9703	34.06272
558	陕西	宝鸡市眉县汤峪镇汤峪村	乡镇	107.8737	34.15014
559	陕西	延安市延川县文安驿镇梁家河村	村庄	110.0596	36.81991
560	陕西	咸阳市泾阳县安吴镇龙源村	村庄	108.8926	34.68386
561	陕西	安康市石泉县后柳镇中坝村	村庄	108.1679	32.92864
562	陕西	商洛市丹凤县棣花镇棣花社区	村庄	110.1968	33.73292
563	陕西	铜川市印台区金锁关镇何家坊村	村庄	109.0077	35.24144

564	陕西	渭南市潼关县太要镇秦王寨社区	村庄	110.3459	34.50762
565	陕西	渭南市临渭区桥南镇天刘村	村庄	109.5983	34.34157
566	陕西	咸阳市旬邑县张洪镇西头村	村庄	108.2149	35.03985
567	陕西	汉中市佛坪县长角坝镇沙窝村	村庄	108.012	33.63649
568	陕西	汉中市汉台区河东店镇花果村	村庄	106.9829	33.20176
569	陕西	安康市宁陕县筒车湾镇七里村	村庄	108.1996	33.42833
570	陕西	铜川市宜君县哭泉镇淌泥河村	村庄	109.0702	35.27991
571	陕西	宝鸡市凤县红花铺镇永生村	村庄	106.7685	34.15817
572	陕西	渭南市华阴市孟塬镇司家村	村庄	110.1475	34.56857
573	陕西	汉中市勉县勉阳街道天荡山社区	地产小区	106.6649	33.18135
574	陕西	商洛市洛南县四皓街道南沟社区	村庄	110.0881	33.96324
575	陕西	杨凌示范区杨陵区五泉镇王上村	村庄	108.0168	34.28618
576	陕西	渭南市华阴市华山镇仙峪口村	村庄	110.0452	34.52284
577	陕西	榆林市绥德县满堂川镇郭家沟村	村庄	110.4146	37.52947
578	陕西	韩城市板桥镇王村	村庄	110.2442	35.52755
579	陕西	商洛市柞水县小岭镇金米村	村庄	109.3034	33.58829
580	甘肃	临夏回族自治州临夏市南龙镇马家庄村	村庄	103.229	35.58704
581	甘肃	陇南市康县王坝镇何家庄村	村庄	105.6634	33.3439
582	甘肃	平凉市泾川县汭丰镇郑家沟村	村庄	107.2486	35.30671
583	甘肃	陇南市康县岸门口镇街道村（朱家沟）	村庄	105.632	33.27406
584	甘肃	兰州市皋兰县什川镇上车村	村庄	103.9992	36.15574
585	甘肃	张掖市肃南裕固族自治县康乐镇榆木庄村	村庄	99.92361	38.97092
586	甘肃	临夏回族自治州临夏县北塬镇钱家村	村庄	103.2175	35.63593
587	甘肃	敦煌市月牙泉镇杨家桥村	村庄	94.66479	40.12287
588	甘肃	张掖市甘州区长安镇前进村	村庄	100.4433	38.90683
589	甘肃	酒泉市肃州区泉湖镇永久村	村庄	98.55801	39.72995
590	甘肃	天水市秦州区玉泉镇李官湾村	村庄	105.7461	34.54529
591	甘肃	庆阳市宁县瓦斜乡永吉村	村庄	107.8883	35.61722
592	甘肃	嘉峪关市峪泉镇黄草营村	村庄	98.1932	39.86912
593	甘肃	甘南藏族自治州迭部县达拉乡高吉村	村庄	103.3143	33.87668
594	甘肃	武威市天祝藏族自治县大红沟镇大红沟村	乡镇	102.5755	37.48278
595	甘肃	金昌市金川区宁远堡镇龙景村	村庄	102.1726	38.43476
596	甘肃	陇南市两当县杨店镇灵官村	村庄	106.3547	33.9417
597	甘肃	甘南藏族自治州迭部县电尕镇谢协村	村庄	103.3381	34.02672
598	甘肃	张掖市山丹县李桥乡高庙村	村庄	101.1178	38.51289
599	甘肃	白银市景泰县喜泉镇大水磅村	村庄	104.1312	36.85147
600	青海	海东市民和回族土族自治县古鄯镇山庄村	村庄	102.6823	36.14857
601	青海	黄南藏族自治州尖扎县昂拉乡德吉村	乡镇	102.0399	35.88969
602	青海	西宁市湟源县和平乡小高陵村	村庄	101.2462	36.60675
603	青海	北藏族自治州门源回族自治县仙米乡桥滩	村庄	102.0478	37.18717
604	青海	海东市循化撒拉族自治县查汗都斯乡红光	村庄	102.2492	35.88145
605	青海	北藏族自治州门源回族自治县珠固乡东旭	村庄	102.2442	37.14401
606	青海	西宁市湟中区拦隆口镇卡阳村	村庄	101.427	36.78483
607	青海	海南藏族自治州贵德县尕让乡松巴村	村庄	101.6507	36.16541
608	青海	西宁市湟中区李家山镇柳树庄村	村庄	101.585	36.72496
609	青海	蒙古族藏族自治州格尔木市郭勒木德镇红	村庄	94.984	36.38969
610	青海	海南藏族自治州贵德县河阴镇红柳滩村	村庄	101.3076	36.05888

611	青海	海东市民和回族土族自治县官亭镇喇家村	村庄	102.8061	35.86853
612	青海	海南藏族自治州贵德县尕让乡二连村	村庄	101.5352	36.12315
613	青海	黄南藏族自治州泽库县和日镇和日村	村庄	101	35.22989
614	青海	玉树藏族自治州治多县立新乡叶青村	村庄	96.17921	33.71115
615	青海	海东市互助土族自治县南门峡镇磨儿沟村	乡镇	101.9023	36.98906
616	青海	北藏族自治州门源回族自治县东川镇麻当	村庄	101.9782	37.26885
617	青海	海北藏族自治州祁连县八宝镇白杨沟村	村庄	100.2556	38.2088
618	青海	海东市互助土族自治县五十镇班彦村	村庄	102.127	36.67208
619	青海	西宁市湟源县申中乡前沟村	村庄	101.2201	36.76371
620	宁夏	银川市西夏区镇北堡镇华西村	乡镇	106.0624	38.62694
621	宁夏	固原市泾源县泾河源镇冶家村	村庄	106.3731	35.39348
622	宁夏	银川市西夏区镇北堡镇昊苑村	乡镇	106.0624	38.62694
623	宁夏	固原市隆德县温堡乡新庄村	村庄	106.0819	35.48604
624	宁夏	吴忠市青铜峡市叶盛镇地三村	村庄	106.1879	38.12151
625	宁夏	银川市贺兰县常信乡四十里店村	村庄	106.358	38.61977
626	宁夏	固原市隆德县神林乡辛平村	村庄	105.8901	35.57272
627	宁夏	吴忠市利通区东塔寺乡石佛寺村	商务大厦	106.2441	38.00306
628	宁夏	中卫市中宁县石空镇倪丁村	村庄	105.6446	37.53839
629	宁夏	固原市西吉县将台堡镇毛沟村	村庄	105.8145	35.81045
630	宁夏	固原市泾源县大湾乡杨岭村	村庄	106.2325	35.77257
631	宁夏	石嘴山市惠农区礼和乡银河村	村庄	106.8298	39.0711
632	宁夏	石嘴山市惠农区红果子镇马家湾村	村庄	106.7011	39.11873
633	宁夏	固原市彭阳县城阳乡杨坪村	村庄	106.8766	35.79962
634	宁夏	固原市原州区河川乡寨洼村	村庄	106.385	35.97496
635	宁夏	吴忠市盐池县花马池镇曹泥洼村	NoClass	107.3184	37.73858
636	宁夏	中卫市中宁县石空镇太平村	村庄	105.6899	37.55908
637	宁夏	固原市隆德县观庄乡前庄村	村庄	106.1495	35.74717
638	宁夏	石嘴山市平罗县黄渠桥镇黄渠桥村	村庄	106.6303	39.01416
639	宁夏	中卫市沙坡头区迎水桥镇北长滩村	村庄	104.7451	37.34604
640	新疆	泰地区喀纳斯景区禾木哈纳斯蒙古族乡哈纟	乡镇	87.4364	48.56958
641	新疆	犁哈萨克自治州新源县那拉提镇阿尔善村	村庄	84.1748	43.30724
642	新疆	郭楞蒙古自治州和静县巴音布鲁克镇巴西	地产小区	84.49553	42.8055
643	新疆	回族自治州木垒哈萨克自治县英格堡乡月	村庄	89.97583	43.75766
644	新疆	阿克苏地区新和县依其艾日克镇加依村	乡镇	82.57441	41.5449
645	新疆	伊犁哈萨克自治州霍城县芦草沟镇四宫村	村庄	80.89687	44.27795
646	新疆	和田地区洛浦县恰尔巴格乡阔恰艾日克村	村庄	80.04837	37.07574
647	新疆	柯尔克孜自治州阿克陶县奥依塔克镇奥	村庄	75.41502	38.96588
648	新疆	巴音郭楞蒙古自治州尉犁县兴平镇达西村	村庄	86.26645	41.34204
649	新疆	喀什地区泽普县国营林场长寿村	旅游景点	76.96738	38.03611
650	新疆	阿克苏地区温宿县柯柯牙镇塔格拉克村	村庄	80.39704	41.68712
651	新疆	阿勒泰地区富蕴县可可托海镇塔拉特村	村庄	89.87456	47.23293
652	新疆	田地区于田县达里雅布依乡达里雅布依	乡镇	82.18331	38.48072
653	新疆	阿克苏地区拜城县康其乡阿热勒村	村庄	81.99858	41.75219
654	新疆	昌吉回族自治州阜康市城关镇山坡中心村	村庄	88.0467	44.12537
655	新疆	楞蒙古自治州博湖县乌兰再格森乡乌图阿	村庄	86.6941	41.9182
656	新疆	郭楞蒙古自治州博湖县才坎诺尔乡拉罕诺	村庄	86.6235	41.92372
657	新疆	喀什地区岳普湖县岳普湖乡喀拉玉吉买村	村庄	76.79148	39.22443

658	新疆	和田地区和田市吉亚乡阔恰村	村庄	80.01543	37.20818
659	新疆	哈萨克自治州昭苏县昭苏镇吐格勒勤布拉	村庄	81.12634	43.15169
660	新疆	喀什地区莎车县米夏镇夏玛勒巴格村	村庄	77.2826	38.43942
661	新疆	昌吉回族自治州吉木萨尔县北庭镇古城村	村庄	89.2195	44.07204
662	新疆	塔拉蒙古自治州温泉县扎勒木特乡博格达	村庄	80.99095	44.97349
663	新疆	地区喀什市帕哈太克里乡尤喀尔克喀库	村庄	75.96691	39.42224
664	新疆兵团	第十二师西山农牧场2连（烽火台小镇）	地产小区	87.38764	43.63825
665	新疆兵团	第十师北屯市185团2连	村庄	87.8449	47.33394
666	新疆兵团	第十师北屯市185团1连	村庄	87.84404	47.33318
667	新疆兵团	第十二师头屯河农场3连	NoClass	87.30967	43.87622
668	新疆兵团	第一师阿拉尔市10团5连	区县	81.27801	40.54684
669	新疆兵团	第八师石河子市121团7连	区县	86.07746	44.30487
670	新疆兵团	第九师161团6连	城市	82.98244	46.21232
671	新疆兵团	第四师可克达拉市76团1连	村庄	80.98698	43.93642
672	新疆兵团	第九师165团4连	城市	84.30343	46.87345
673	新疆兵团	第五师双河市83团1连	区县	82.36855	44.84617
674	新疆兵团	第四师可克达拉市71团7连	村庄	80.98775	43.93726
675	新疆兵团	第十三师红星一场3连	购物	93.59809	42.78015
676	新疆兵团	第十师北屯市188团4连	村庄	87.67533	47.32722
677	新疆兵团	第五师双河市86团22连	政府机构	82.14788	44.8912
678	新疆兵团	第十三师红星二场3连	购物	93.29845	42.89374
679	新疆兵团	第四师可克达拉市62团3连	村庄	80.99251	43.93234
680	新疆兵团	第十二师104团畜牧连	NoClass	87.43324	43.67411
