# Supplementary material for: The spatial distribution characteristics and influencing factors of key villages in rural tourism in China
Source: PLoS One. 2025 Aug 19;20(8):e0330486. doi: 10.1371/journal.pone.0330486 (PMC12364373; doi:10.1371/journal.pone.0330486)
Supplement: S4 File — (PDF) [file pone.0330486.s004.pdf]

ID	省份	5A景区名称	经度	纬度
1	北京	北京市海淀区圆明园景区	116.284673	40.01963322
2		北京市奥林匹克公园	116.3869185	39.99949021
3		恭王府景区	116.3801291	39.93520477
4		北京市明十三陵景区	116.219132	40.2566219
5		北京八达岭－慕田峪长城旅游区	115.9717428	40.3610917
6		颐和园	116.2662954	39.99731352
7		天坛公园	116.4045942	39.8805411
8		故宫博物院	116.3908029	39.91634784
9	天津	天津盘山风景名胜区	117.2758877	40.09592228
10		天津古文化街旅游区（津门故里）	117.1862549	39.14274701
11	河北	河北省承德市金山岭长城景区	117.2411229	40.69830964
12		河北省保定市清西陵景区	115.3383443	39.34000653
13		秦皇岛市山海关景区	119.7550979	40.00888655
14		河北省保定市白石山景区	114.6966997	39.2157765
15		河北省邯郸市广府古城景区	114.7245004	36.7008246
16		邯郸市娲皇宫景区	113.602415	36.63376276
17		唐山市清东陵景区	117.6777311	40.17390293
18		河北省石家庄市西柏坡景区	113.9377736	38.33980493
19		河北保定野三坡景区	116.4007584	39.90319643
20		保定市安新白洋淀景区	115.9838638	38.93567814
21		承德避暑山庄及周围寺庙景区	118.1675529	40.76667071
22	山西	黄河壶口瀑布旅游区（陕西省延安市·山西省临汾市）	111.5127819	36.08802389
23		山西省临汾市云丘山景区	111.0150614	35.7535932
24		山西省长治市壶关太行山大峡谷八泉峡景区	113.5715047	35.91810269
25		山西省临汾市洪洞大槐树寻根祭祖园景区	111.6695195	36.27141273
26		山西省忻州市雁门关景区	112.8764173	39.1833471
27		晋中市平遥古城景区	112.1772971	37.20271137
28		晋中市介休绵山景区	111.9863242	36.88907057
29		山西晋城皇城相府生态文化旅游区	116.6692243	39.84541256
30		忻州市五台山风景名胜区	113.5825958	38.96987473
31		大同市云冈石窟	113.130685	40.11182334
32	蒙古自治	内蒙古自治区阿拉善盟胡杨林旅游区	101.0782109	41.95995265
33		内蒙古自治区赤峰市阿斯哈图石阵旅游区	118.8828299	42.253765
34		内蒙古自治区阿尔山·柴河旅游景区	120.2821328	46.96478382
35		内蒙古自治区满洲里市中俄边境旅游区	117.3522853	49.62742711
36		内蒙古鄂尔多斯成吉思汗陵旅游区	116.3147308	39.96928441
37		内蒙古鄂尔多斯响沙湾旅游景区	116.3147308	39.96928441
38	辽宁省	辽宁省盘锦市红海滩风景廊道景区	121.8402416	40.90050192
39		辽宁省鞍山市千山景区	123.1094275	41.08479049
40		本溪市本溪水洞景区	124.0616389	41.30099028
41		辽宁大连金石滩景区	122.0105867	39.08967125
42		大连老虎滩海洋公园.老虎滩极地馆	121.6722476	38.87764523
43		沈阳市植物园	116.4919404	39.98296619
44	吉林省	吉林省通化市高句丽文物古迹旅游景区	126.1562402	41.16555646
45		吉林省长春市世界雕塑公园旅游景区	125.3271012	43.82014428
46		敦化市六鼎山文化旅游区	128.2385538	43.32131905

47		长春市长影世纪城旅游区	125.2896097	43.85990184
48		吉林长春净月潭景区	125.4698214	43.77690096
49		长春市伪满皇宫博物院	125.3420883	43.90335289
50		长白山景区	116.1855565	39.87175635
51	黑龙江	黑龙江省虎林市虎头旅游景区	133.6612533	45.98291702
52		漠河北极村旅游区	116.1864762	39.85102713
53		伊春市汤旺河林海奇石景区	129.4496478	48.4589177
54		黑龙江牡丹江镜泊湖景区	128.9462353	44.0586292
55		黑龙江黑河五大连池景区	126.1553734	48.69122568
56		哈尔滨市太阳岛景区	126.5560707	45.80676349
57	上海	上海市中国共产党一大·二大·四大纪念馆景区	121.4804414	31.25712324
58		上海科技馆	116.4007584	39.90319643
59		上海野生动物园	116.329184	39.49597884
60		上海东方明珠广播电视塔	121.4953353	31.24178797
61	江苏省	江苏省宿迁市洪泽湖湿地景区	118.3275165	33.26368395
62		江苏省无锡市惠山古镇景区	120.2717419	31.58413868
63		江苏省常州市中国春秋淹城旅游区	119.9190554	31.7045065
64		江苏省连云港花果山景区	115.609525	39.64200591
65		江苏省徐州市云龙湖景区	117.1472131	34.23956164
66		大丰中华麋鹿园景区	120.8109879	32.98575761
67		周恩来故里旅游景区	116.4007584	39.90319643
68		镇江市句容茅山景区	119.2986551	31.78717503
69		常州市天目湖景区	119.4403221	31.32047866
70		苏州市沙家浜·虞山尚湖旅游区	120.681891	31.64255101
71		苏州市吴中太湖旅游区	120.4398661	31.22079191
72		无锡市鼋头渚景区	120.2223951	31.5254395
73		镇江市金山·焦山·北固山旅游景区	119.4519734	32.21868165
74		苏州市金鸡湖景区	120.6970416	31.30627258
75		江苏省姜堰市溱湖旅游景区	120.0813786	32.61431434
76		南通市濠河景区	120.862769	32.0164726
77		扬州市瘦西湖风景区	119.4161529	32.41050933
78		常州市环球恐龙城休闲旅游区	119.9948217	31.82374882
79		南京市夫子庙-秦淮风光带景区	118.7843627	32.02195316
80		苏州市同里古镇景区	120.7193742	31.15664243
81		无锡市灵山景区	120.0974113	31.43069429
82		中央电视台无锡影视基地三国水浒景区	120.2286071	31.47911114
83		南京市钟山风景名胜区-中山陵园风景区	118.8477268	32.06579424
84		苏州市周庄古镇景区	120.8476314	31.11585063
85		苏州园林（拙政园、虎丘山、留园）	116.3380549	39.94878569
86	浙江省	浙江省台州市台州府城文化旅游区	121.1128313	28.84940893
87		浙江省温州市刘伯温故里景区	119.9293627	27.8503221
88		浙江省丽水市缙云仙都景区	120.1079803	28.70307593
89		浙江省宁波市天一阁·月湖景区	121.5397618	29.87070275
90		浙江省衢州市江郎山·廿八都景区	118.4782435	28.29547391
91		浙江省嘉兴市西塘古镇旅游景区	120.8939286	30.94976723
92		台州市神仙居景区	120.5991598	28.69152667
93		台州市天台山景区	121.031146	29.15965346

94		湖州市南浔古镇景区	120.424186	30.870921
95		衢州市开化根宫佛国文化旅游景区	118.3877535	29.13628911
96		浙江省绍兴市鲁迅故里沈园景区	120.5859446	29.99159944
97		浙江省杭州西溪湿地旅游区	120.1448301	30.27738896
98		浙江省嘉兴市南湖旅游区	120.7542017	30.75391931
99		金华市东阳横店影视城景区	120.312417	29.14057503
100		宁波市奉化溪口-滕头旅游景区	121.2547124	29.68716165
101		嘉兴市桐乡乌镇古镇旅游区	120.4874162	30.75017968
102		杭州市千岛湖风景名胜区	115.7691892	40.4617142
103		舟山市普陀山风景名胜区	122.3868728	30.00389753
104		温州市雁荡山风景名胜区	121.029455	28.37707919
105		杭州市西湖风景名胜区	120.1167521	30.2248617
106	安徽省	安徽省马鞍山市长江采石矶文化生态旅游区	118.4539529	31.65241429
107		安徽省六安市万佛湖景区	116.7719085	31.3064579
108		安徽省芜湖市方特旅游区	118.3705359	31.3795945
109		合肥市三河古镇景区	117.2394583	31.5135854
110		黄山市古徽州文化旅游区	118.3330813	29.83449733
111		阜阳市颍上八里河景区	116.3025398	32.56371872
112		安徽省宣城市绩溪龙川景区	118.6652601	30.07928746
113		六安市天堂寨旅游景区	115.7735325	31.13713622
114		安徽省黄山市皖南古村落—西递宏村	117.9679263	29.89484416
115		安徽省安庆市天柱山风景区	116.4549167	30.73851414
116		池州市九华山风景区	117.818805	30.48119411
117		黄山市黄山风景区	118.1562312	30.1269161
118	福建省	福建省莆田市湄洲岛妈祖文化旅游区	119.1241286	25.06602555
119		龙岩市古田旅游区	116.8191437	25.22644656
120		福州市三坊七巷景区	119.2914003	26.08983968
121		宁德市福鼎太姥山旅游区	120.2473812	27.1028748
122		泉州市清源山景区	118.6073177	24.94838383
123		宁德市白水洋-鸳鸯溪旅游区	119.1109171	27.05619486
124		福建省土楼（永定・南靖）旅游	117.0012965	24.6640537
125		福建省三明市泰宁风景旅游区	117.0515671	26.84682479
126		南平市武夷山风景名胜区	117.9516847	27.66510408
127		厦门市鼓浪屿风景名胜区	115.7691892	40.4617142
128	江西省	江西省赣州市三百山景区	115.4202849	25.00091293
129		江西省九江市庐山西海景区	116.3704364	39.94540484
130		江西省萍乡市武功山景区	114.1240741	27.4921175
131		江西省南昌市滕王阁旅游区	115.8772156	28.68121497
132		江西省上饶市龟峰景区	117.4209586	28.32081858
133		江西省抚州市大觉山景区	117.1384275	27.70102106
134		宜春市明月山旅游区	114.2873406	27.60297846
135		瑞金市共和国摇篮旅游区	116.0221278	25.88836697
136		景德镇古窑民俗博览区	116.4007584	39.90319643
137		上饶市婺源江湾景区	118.0462106	29.37068451
138		江西省鹰潭市龙虎山旅游景区	116.9764573	28.12255803
139		江西省上饶市三清山旅游景区	117.9761633	28.45076003
140		吉安市井冈山风景旅游区	114.1319981	26.63322542

141		江西省庐山风景名胜区	115.9894388	29.5583931
142	山东省	山东省济宁市微山湖旅游区	117.2705822	34.6847616
143		山东省临沂市萤火虫水洞・地下大峡谷旅游区	118.535028	35.72841438
144		山东省东营市黄河口生态旅游区	119.0701514	37.80291224
145		山东省威海市华夏城旅游景区	122.1105498	37.42322591
146		山东省潍坊市青州古城旅游区	118.4734276	36.68042509
147		山东省沂蒙山旅游区	116.4007584	39.90319643
148		济南市天下第一泉景区	116.3733946	39.87115031
149		枣庄市台儿庄古城景区	117.7373067	34.55879464
150		山东烟台龙口南山景区	120.4744686	37.559998
151		山东威海刘公岛景区	122.1858691	37.50339313
152		山东青岛崂山景区	120.6146004	36.18451087
153		济宁市曲阜明故城（三孔）旅游区	116.98505	35.59684363
154		烟台市蓬莱阁旅游区(三仙山-八仙过海)	120.7711089	37.82472517
155		泰安市泰山景区	117.1289377	36.19297533
156	河南省	河南省信阳市鸡公山景区	114.0627053	32.13332811
157		河南省新乡市八里沟景区	113.5364049	35.58795085
158		河南省永城市芒砀山旅游景区	116.5060364	34.1707731
159		河南省红旗渠・太行大峡谷	116.4007584	39.90319643
160		驻马店市嵖岈山旅游景区	113.7240666	33.13145753
161		南阳市西峡伏牛山老界岭・恐龙遗址园旅游区	111.6805468	33.23215033
162		洛阳市龙潭大峡谷景区	111.9878451	34.96149115
163		河南省洛阳栾川老君山・鸡冠洞旅游区	111.5647121	33.78494003
164		河南省平顶山市尧山-中原大佛景区	112.454039	33.76652077
165		河南开封清明上河园	116.4007584	39.90319643
166		河南洛阳白云山景区	111.3870321	33.91100834
167		河南安阳殷墟景区	114.4644121	36.0822553
168		焦作市云台山一神农山・青天河景区	113.0047031	35.34156132
169		洛阳市龙门石窟景区	112.4695132	34.55076633
170		登封市嵩山少林景区	112.9462752	34.51569366
171	湖北省	湖北省宜昌市三峡大瀑布景区	111.3167354	30.92732067
172		湖北省恩施州腾龙洞景区	108.981604	30.33212295
173		湖北省襄阳市古隆中景区	111.6638422	32.38099231
174		湖北省咸宁市三国赤壁古战场景区	113.6250759	29.88156473
175		恩施州恩施大峡谷景区	109.1619807	30.42831882
176		武汉市黄陂木兰文化生态旅游区	114.3911273	31.09529062
177		武汉市东湖景区	114.4078949	30.55448888
178		宜昌市长阳清江画廊景区	111.2354734	30.53102728
179		湖北省神农架旅游区	116.4007584	39.90319643
180		湖北省恩施州神农溪纤夫文化旅游区	116.6692243	39.84541256
181		湖北省十堰市武当山风景区	111.0576056	32.46346107
182		湖北省宜昌市三峡人家风景区	111.1501592	30.79294738
183		宜昌市三峡大坝-屈原故里旅游区	110.8245108	30.93239712
184		武汉市黄鹤楼公园	114.3001689	30.54602077
185	湖南省	省湘西土家族苗族自治州矮寨・十八洞・德夯大峡谷	109.5826851	28.3426242
186		湖南省常德市桃花源旅游区	111.436133	28.79362315
187		湖南省株洲市炎帝陵景区	113.6670047	26.42611609

188		湖南省邵阳市崀山景区	110.7908412	26.3575034
189		郴州市东江湖旅游区	113.3054846	25.86897675
190		长沙市花明楼景区	112.6386372	28.0417904
191		湖南省长沙市岳麓山·橘子洲旅游区	112.932067	28.18804521
192		湖南省岳阳市岳阳楼—君山岛景区	113.1240359	29.37454822
193		湖南省湘潭市韶山旅游区	112.4883769	27.91132158
194		衡阳市南岳衡山旅游区	112.7100678	27.27310042
195		张家界武陵源—天门山旅游区	110.4918058	29.01521096
196	广东省	广东省江门市开平碉楼文化旅游区	112.5746269	22.37559769
197		广东省肇庆市星湖旅游景区	112.4641651	23.05720001
198		广东省惠州市惠州西湖旅游景区	114.4020997	23.08757672
199		广东省中山市孙中山故里旅游区	113.5230137	22.4453471
200		阳江市海陵岛大角湾海上丝路旅游区	111.8424256	21.5719956
201		佛山市长鹿旅游休博园	113.283467	22.88086529
202		惠州市罗浮山景区	114.0636364	23.26531209
203		佛山市西樵山景区	112.971982	22.92741649
204		广东省韶关市丹霞山景区	113.7157943	25.02457401
205		广东省清远市连州地下河旅游景区	112.3246439	25.00452604
206		深圳市观澜湖休闲旅游区	113.9905512	22.69873926
207		梅州市雁南飞茶田景区	116.36016	24.38938483
208		广东省广州市白云山风景区	113.2907931	23.16310021
209		深圳华侨城旅游度假区	113.9797785	22.54108532
210		广州市长隆旅游度假区	113.3198906	23.00270269
211	西壮族自	广西壮族自治区贺州市黄姚古镇景区	111.2015948	24.25048722
212		广西壮族自治区北海市涠洲岛南湾鳄鱼山景区	109.0980079	21.01175354
213		广西壮族自治区百色市百色起义纪念园景区	106.636224	23.90476295
214		广西壮族自治区崇左市德天跨国瀑布景区	106.7248384	22.85587759
215		广西自治区桂林市两江四湖·象山景区	110.2914423	25.27156986
216		南宁市青秀山旅游区	108.3464216	22.86535865
217		桂林市独秀峰-王城景区	110.2946233	25.28437538
218		桂林市乐满地度假世界	110.2987591	25.303145
219		桂林市漓江景区	110.4269964	25.14989764
220	海南省	海南省三亚市蜈支洲岛旅游区	109.7622052	18.31215073
221		海南槟榔谷黎苗文化旅游区	116.6692243	39.84541256
222		分界洲岛旅游区	116.4007584	39.90319643
223		海南呀诺达雨林文化旅游区	116.6692243	39.84541256
224		三亚市南山大小洞天旅游区	109.152842	18.30424968
225		三亚市南山文化旅游区	109.2048467	18.30050806
226	重庆	重庆市奉节县白帝城·瞿塘峡景区	109.5755274	31.04080264
227		重庆市黔江区濯水景区	108.7679514	29.29730643
228		重庆市彭水县阿依河景区	108.1160743	29.15345389
229		重庆市云阳龙缸景区	108.9999429	30.66792674
230		江津四面山景区	106.3869273	28.6195248
231		重庆市南川金佛山	107.0997279	29.15526757
232		重庆市万盛经开区黑山谷景区	106.922798	28.96836471
233		酉阳桃花源旅游景区	108.7671532	28.85158026
234		武隆喀斯特旅游区(天生三桥·仙女山·芙蓉洞)	107.8067609	29.43627584

235		重庆巫山小三峡-小小三峡	109.8835216	31.08280652
236		重庆大足石刻景区	105.7924612	29.75214268
237	四川省	四川省成都市安仁古镇景区	103.6219463	30.50790374
238		四川省甘孜州稻城亚丁旅游景区	116.4007584	39.90319643
239		四川省巴中市光雾山旅游景区	106.7965601	32.69072365
240		四川省雅安市碧峰峡旅游景区	102.9887708	30.0751693
241		四川省甘孜州海螺沟景区	116.4007584	39.90319643
242		四川省南充市仪陇朱德故里景区	106.5979736	31.46478029
243		广元市剑门蜀道剑门关旅游区	105.5737592	32.22649666
244		广安市邓小平故里旅游区	106.6329003	30.52222027
245		南充市阆中古城旅游区	105.9675059	31.57683813
246		阿坝州汶川特别旅游区	103.4861818	31.05773421
247		绵阳市北川羌城旅游区	104.4611749	31.60979904
248		四川省阿坝州黄龙景区	109.8348155	35.58479032
249		乐山市乐山大佛景区	103.772281	29.54115301
250		阿坝藏族羌族自治州九寨沟旅游景区	102.2233554	31.90154632
251		乐山市峨眉山景区	103.3475961	29.57043775
252		成都市青城山--都江堰旅游景区	103.5928479	30.90217708
253	贵州省	贵州省毕节市织金洞景区	105.8984435	26.77226629
254		贵州省遵义市赤水丹霞旅游区	105.9449996	28.40362073
255		贵州省黔东南州镇远古城旅游景区	108.4200918	27.05331226
256		贵州省铜仁市梵净山旅游区	109.1905138	27.7410269
257		贵州省贵阳市花溪青岩古镇景区	106.6825028	26.33516644
258		黔南州荔波樟江景区	107.7431602	25.25265295
259		毕节市百里杜鹃景区	105.9351973	27.18317501
260		安顺市龙宫景区	105.8819856	26.11007142
261		安顺市黄果树大瀑布景区	105.6704368	25.98048428
262	云南省	云南省文山州普者黑旅游景区	116.4007584	39.90319643
263		云南省保山市腾冲火山热海旅游区	98.44014882	24.95007905
264		云南省昆明市昆明世博园景区	102.7571213	25.07965165
265		迪庆州香格里拉普达措景区	99.94358458	27.8622693
266		中国科学院西双版纳热带植物园	116.5878322	39.6579966
267		大理市崇圣寺三塔文化旅游区	116.6692243	39.84541256
268		丽江市丽江古城景区	100.2248659	26.88064137
269		丽江市玉龙雪山景区	100.2028757	27.1372919
270		昆明市石林风景区	103.3216953	24.81420841
271	西藏自治区	林芝市雅鲁藏布大峡谷旅游景区	94.93721365	29.61586167
272		日喀则扎什伦布寺景区	116.4007584	39.90319643
273		林芝巴松措景区	116.4007584	39.90319643
274		拉萨市大昭寺	116.3839451	39.98190669
275		拉萨布达拉宫景区	116.4007584	39.90319643
276	陕西省	黄河壶口瀑布旅游区（陕西省延安市·山西省临汾市）	111.5127819	36.08802389
277		陕西省西安市大明宫旅游景区	108.95919	34.31359824
278		陕西省延安市延安革命纪念地景区	109.4833305	36.57665633
279		陕西省西安市城墙·碑林历史文化景区	108.9435694	34.25321786
280		陕西省宝鸡市太白山旅游景区	107.904241	34.13454827

281		商洛市金丝峡景区	110.5615364	33.35637802
282		宝鸡市法门寺佛文化景区	107.8971237	34.43618182
283		陕西渭南华山景区	110.0611165	34.49271551
284		陕西西安大雁塔·大唐芙蓉园景区	108.9695257	34.21423558
285		延安市黄帝陵景区	109.2645575	35.58589789
286		西安市华清池景区	116.366951	39.92568018
287		西安市秦始皇兵马俑博物馆	109.273463	34.38496165
288	甘肃省	甘肃省陇南市官鹅沟景区	104.3899105	34.01147223
289		甘肃省临夏州炳灵寺世界文化遗产旅游区	116.4007584	39.90319643
290		甘肃省张掖市七彩丹霞景区	100.0676373	38.95738118
291		敦煌鸣沙山月牙泉景区	94.67772138	40.08795555
292		甘肃天水麦积山景区	105.9981856	34.36652206
293		平凉市崆峒山风景名胜区	106.4814572	35.53663309
294		嘉峪关市嘉峪关文物景区	98.22620009	39.80326222
295	青海省	青海省海北州阿咪东索景区	116.4007584	39.90319643
296		青海省海东市互助土族故土园景区	101.9351647	36.83426874
297		西宁市塔尔寺景区	101.5661795	36.48814266
298		青海省青海湖景区	116.4007584	39.90319643
299	宁夏回族自治区	银川市灵武水洞沟旅游区	106.5043905	38.28995343
300		宁夏银川镇北堡西部影视城	116.4007584	39.90319643
301		中卫市沙坡头旅游景区	104.9986469	37.47658061
302		石嘴山市沙湖旅游景区	106.3569747	38.80098671
303	新疆维吾尔自治区	新疆维吾尔自治区昌吉回族自治州江布拉克景区	89.68514478	43.54980051
304		新疆生产建设兵团阿拉尔市塔克拉玛干·三五九旅文化旅	116.3185996	39.88060627
305		新疆生产建设兵团第十师白沙湖景区	116.3185996	39.88060627
306		新疆维吾尔自治区博尔塔拉蒙古自治州赛里木湖景区	81.18484995	44.49421753
307		新疆维吾尔自治区克拉玛依市世界魔鬼城景区	85.74466739	46.12978122
308		新疆维吾尔自治区喀什地区帕米尔旅游区	75.19923728	37.79525275
309		新疆自治区巴音州和静巴音布鲁克景区	116.2879307	39.8720609
310		新疆自治区伊犁州喀拉峻景区	116.4007584	39.90319643
311		喀什地区喀什噶尔老城景区	75.98593458	39.47243099
312		巴音郭楞蒙古自治州博斯腾湖景区	86.77616975	41.74397658
313		乌鲁木齐天山大峡谷景区	87.43844187	43.49178888
314		喀什地区泽普金湖杨景区	116.4342849	39.90866718
315		阿勒泰地区富蕴可可托海景区	89.87957386	47.22095703
316		新疆伊犁那拉提旅游风景区	116.4007584	39.90319643
317		阿勒泰地区喀纳斯景区	87.12855077	48.50624287
318		吐鲁番市葡萄沟风景区	89.24030476	42.99795333
319		新疆天山天池风景名胜区	115.7691892	40.4617142
